# Impact of a GABA‐Producing *Lactococcus lactis* on Microbiota and Mycobiota During CNS Inflammatory Demyelination

**DOI:** 10.1096/fba.2025-00082

**Published:** 2026-01-07

**Authors:** Kristina Hill, Alexandra LaFollette, Trevor O. Kirby, Soledad Negrete, Dani Babcock, Kari Felton, Hannah Kohl, Kavita Sharma, Andrea Castillo, Jean‐Baptiste Roullet, K. Michael Gibson, Javier Ochoa‐Repáraz

**Affiliations:** ^1^ Biomolecular Sciences Graduate Program Boise State University Boise Idaho USA; ^2^ Department of Biological Sciences Boise State University Boise Idaho USA; ^3^ AG1, Research, Nutrition, and Innovation Carson City Nevada USA; ^4^ Department of Biology Eastern Washington University Cheney Washington USA; ^5^ Department of Biomedical and Pharmaceutical Sciences Idaho State University Pocatello Idaho USA; ^6^ Department of Pharmacotherapy, College of Pharmacy and Pharmaceutical Sciences Washington State University Spokane Washington USA

**Keywords:** EAE, GABA, *Lactococcus lactis*, microbiota, mycobiota, proteomics

## Abstract

Gut microbes are key regulators of immune homeostasis. Their composition fluctuates over time and between individuals and is also influenced by disease. We and others have reported changes in gut bacterial composition following induction of experimental autoimmune encephalomyelitis (EAE), a well‐established model for multiple sclerosis (MS). Specifically, we observed reductions in the abundance of bacteria capable of producing gamma‐aminobutyric acid (GABA). Because GABA regulates immune cell function, we genetically engineered a *Lactococcus lactis* strain to overproduce GABA (P8s‐GAD *L. lactis* ) and hypothesized that this strain would have protective activity in EAE. To test this hypothesis, a suspension of P8s‐GAD *L. lactis* was administered by gavage to C57BL/6 Envigo (Env) and Jackson Laboratories (Jax) mice at the time of EAE induction. Controls included mice treated with unmodified *L. lactis (P‐L. lactis)* and mice treated with sterile bacterial medium. P8s‐GAD *L. lactis* was clinically protective in Env mice but not in Jax mice. To understand the lack of protection in Jax mice, we examined the effects of treatments on intestinal micro‐ and mycobiota using 16S rRNA and IST sequencing, and samples were collected at disease induction, 14 days after, and at the end of the experiment (day 28). We also examined the impact of treatments on the brain, using whole‐brain proteomics (day 28). Despite the lack of disease protection, P8s‐GAD *L. lactis* significantly modified the gut microbiome by affecting broad taxonomic composition, as quantified by beta‐diversity changes over time, and the CNS protein profile, including an increase in Gabra6 expression, the alpha‐6 subunit of the GABA type A (GABA_R_A) receptor. These changes, combined with reduced EAE severity observed in Env mice, suggest that GABA‐producing bacteria could be considered for the treatment of neuroinflammatory conditions. The study also highlights the importance of controlling the mouse source in probiotic and microbiota research within experimental models of immune‐mediated diseases.

## Graphical abstract

GABA‐producing *Lactococcus lactis* protects against experimental autoimmune encephalomyelitis (EAE) in a mouse source‐dependent manner. The study focuses on the therapeutic potential of genetically engineered gamma‐aminobutyric acid (GABA)‐overproducing *Lactococcus lactis* (P8s‐GAD *L. lactis* ) in the EAE model. EAE was induced in C57BL/6 mice from two different commercial sources: Envigo (Env) and Jackson Laboratories (Jax). Mice were orally treated with P8s‐GAD *L. lactis* , unmodified *L. lactis* (P‐ *L. lactis* ), or sterile medium. Clinical scores (EAE severity) were monitored. Gut microbiota/mycobiota were analyzed via 16S rRNA/ITS sequencing, and whole‐brain proteomics were performed.

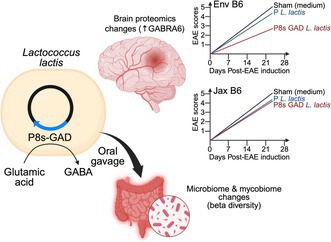

## 1 Introduction

There is increasing interest in the role of the gut microbiome in modulating a wide range of human autoimmune, neuroinflammatory, and neurobehavioral disorders. The experimental model, experimental autoimmune encephalomyelitis (EAE), is the most widely used CNS inflammatory demyelination model of multiple sclerosis (MS). We previously reported that EAE induction leads to dysbiosis [1, 2, 3]. In MS patients, the gut microbiota composition differs from that of healthy individuals [4, 5, 6, 7, 8, 9, 10]. Among the wide range of possible effects of the gut‐microbiota‐brain axis, neuroinflammation triggered during EAE reduced the relative abundances of gamma‐aminobutyric acid (GABA)‐producing bacteria [1, 2, 3]. GABA levels are reduced in the brain and blood of MS patients [11, 12, 13, 14], and GABA and GABAergic compounds are protective against EAE [15]. Moreover, GABA exposure to immune cells promotes immunomodulatory responses [15, 16, 17, 18].

Because of the gut microbiome's importance in regulating the immune system and modulating human diseases, probiotics can be administered to alleviate disease. We successfully engineered a GABA‐producing *Lactococcus lactis* strain to study the impact of gut‐produced GABA on EAE progression. *L. lactis* is a lactic acid bacterium (LAB), generally regarded as safe (GRAS), that has been extensively evaluated as an oral delivery vector of active inducers or inhibitors of immune responses and immune response inhibitors [19]. We genetically modified *L. lactis* to enhance its GABA‐synthesizing enzymatic machinery. This *L. lactis* IL1408 strain contained a plasmid with an extra copy of the *gadCB* operon, regulated by a strong constitutive *L. lactis* promoter (P8s) [20]; *gadB* encodes glutamic acid decarboxylase, which converts glutamic acid to GABA. Our published *L. lactis* ‐P8s‐GAD, with constitutive *gadCB* expression that does not require an exogenous inducer [20], is poised for development as a therapeutic for neuroinflammation and other neurological and peripheral diseases [21].

Although there is an increasing understanding of the biological relevance of the microbiome to the host, the underlying mechanisms of the gut‐brain axis remain to be fully elucidated. In EAE, the onset of the disease causes significant microbiome changes; in turn, these changes impact disease outcomes [22, 23, 24]. However, the temporal relationship between dysbiosis and neuroinflammation is unknown, as is the impact of microbiota composition on therapeutic strategies. We found that C57BL/6 mice obtained from different vendors, Envigo (Env) and Jackson Laboratories (Jax), had distinct microbiome makeup upon arrival, namely, significant differences in alpha and beta diversity and in taxon abundances [2]. Jax mice had an overabundance of Actinobacteria and Verrucomicrobiota, including members of *Akkermansia*, compared to Env mice at the time of EAE induction. Furthermore, disease induction led to distinct microbiome modifications in both Env and Jax mice, as indicated by strain differences in diversity and taxa abundances on day 14 post‐EAE induction. At day 21, the end of the experimental period, Jax mice showed increased abundance of Actinobacteria and decreased abundance of Verrucomicrobiota compared to Env mice. Strain‐specific changes in diversity and taxa profile were associated with different clinical outcomes, suggesting that baseline microbiome composition influences disease severity [2]. Of relevance to the present report is the observation that disease progression in Jax mice was associated with reduced relative abundance of taxa of the LAB‐containing order Lactobacillales [2]. In a more recent study, we reported that microbiome differences between Env and Jax mice were also observed at the functional level [25].

In this current project, we treated Env and Jax EAE mice with our GABA‐producing *L. lactis* ‐P8s‐GAD strain. The treatment reduced EAE severity in Env mice but not in Jax mice. Despite the lack of protection, oral administration of P8s‐GAD *L. lactis* significantly altered gut microbial composition, primarily in the bacterial fraction, with modest effects on the mycobiota. The addition of the GABA metabolic precursor, glutamic acid, did not result in clinical protection. However, it impacted both the gut microbiome and mycobiome and modified brain protein composition, as assessed by whole‐brain proteomics. Our data support previous studies suggesting that the host's microbial makeup is an important factor in regulating the outcomes of immune‐mediated experimental treatments.

## 2 Materials and Methods

### 2.1 Animals and Housing Conditions

Ten‐week‐old female C57BL6 NHsd mice (Envigo RMS Inc., Indianapolis, IN, USA; RRID:MGI:2161078; Env) and C57BL6 Jax mice (Jackson Laboratories, Bar Harbor, ME, USA; RRID:IMSR_JAX:000664; Jax) were randomly housed in groups of 5 in wire‐top cages with a 12‐h light/dark cycle (22°C ± 1°C; 23%–33% humidity). All animals weighed approximately 20 g. Mice had free access to food and water, with all care and procedures following Eastern Washington University (EWU) and Boise State University (BSU) institutional policies for animal well‐being and health. Teklad 22/5 rodent diet (Envigo RMS) was used in Env mice, while Laboratory Rodent Diet 5001 (LabDiet Inc., Richmond, IN) was used in experiments with Jax mice at BSU. Mice had free access to food and water and were housed and handled ethically, in accordance with approved Institutional Animal Care and Use Committee (IACUC) protocols for animal well‐being and health, under IACUC protocols 2018‐11‐01 (EWU) and AC22‐018 (BSU). Each experimental group consisted of 10 mice (*n* = 10). The sample size for the EAE experiments was determined based on a power analysis using previous ANOVA findings (*p* = 0.045 for day 25 vs. untreated; treatment vs. control) at a desired power of 90% and a significance level of 0.05.

### 2.2 EAE Induction and Clinical Score Evaluation

Animals were given a week to acclimate to the housing environments. EAE was induced using the EAE induction kit from Hooke Laboratories (Hooke Kit EK‐2110, Hooke Laboratories, Lawrence, MA). The kits use MOG_35‐55_ in emulsion with complete Freund's adjuvant (CFA) and Pertussis toxin (PTX) in a glycerol buffer. On day 0 of EAE induction, the MOG_35–55_‐CFA emulsion was diluted with PTX in phosphate‐buffered saline and injected subcutaneously. PTX toxin injection was repeated the following day (day +1). Mice were scored under blinded observations as described by us and others: 0—no detectable signs of EAE, 0.5—distal limp tail, 1.0—complete limp tail, 1.5—limp tail and hind limb weakness, 2.0—unilateral partial hind limb paralysis, 2.5—bilateral partial hind limb paralysis, 3.0—complete bilateral hind limb paralysis, 3.5—complete bilateral hind limb paralysis and partial front limb paralysis, 4.0—quadriplegia, 5—dead animal. Attrition: mice that were found dead received a clinical score of 5. Losses not caused by EAE induction were not observed.

### 2.3 Bacterial Growth, Dosing Preparations, and Treatments

*Lactococcus lactis* ‐P8s‐GAD and *Lactococcus lactis* ‐P (unmodified strain, carrying plasmid without engineered construct) strains were cultured in M17 broth (BD Difco, USA) or agar (Thermo Scientific Chemicals, USA) with 0.5% glucose (Sigma‐Aldrich, USA) and 5 mg mL^−1^ erythromycin (GM17 erm media) at 30°C without aeration overnight [20]. After culturing for 18 h, the strains were diluted to an OD_600_ of 0.2 in GM17 or GM17 erm and incubated at 30°C for 3 h. At the time of sample collection, the number of bacterial cells was determined using a spectrophotometer (OD_600_). Viable plate count experiments were previously used to correlate cell number with spectrophotometric absorbance at OD_600_ [20].

Env and Jax mice were treated five consecutive days per week with oral gavage with 5 × 10^8^ CFU/mouse of P‐ *L. lactis* (P) or P8s‐GAD *L. lactis* (P8s) suspended in sterile saline. Treatments started either on the day of EAE induction (Day 0) or 7 days before EAE induction (Day −7) and continued until Day 28. Treatment protocols, including dosing, are shown in all relevant legend figures. In some experiments, the GM17 medium used for bacterial administration was supplemented with 200 mM glutamic acid hydrochloride (Sigma, G2128). In other experiments, glutamic acid hydrochloride (200 mM) was provided in the drinking water. All animals had free access to water.

### 2.4 Stool Sample Collections and 16S Ribosomal RNA (rRNA) and ITS Sequencing

Stool samples were collected in sterile tubes on days −7, 0, 14, and 28 post‐EAE induction (dpi) and stored at −80°C before analysis. Seven animals per group (*n* = 7) were used for microbiome and mycobiome stool analysis. Samples were sent to the University of Wyoming for DNA extraction and sequencing. Qiagen DNA stool extraction kits were used for DNA isolation. DNA aliquots (1 ng/mL DNA) were analyzed by PCR using primers specific to the variable regions 3 and 4 (V3–V4) of the prokaryotic 16S rRNA gene and the fungal internal transcribed spacer (ITS) gene. Library preparation and V3‐V4 amplicon sequencing were performed on the Illumina MiSeq (RRID: SCR_016379) platform. A modified protocol with the Nextera XT kit was used for library preparation, and sequencing was performed using MiSeq V2 (2 × 250 bp) chemistry. The sequencing protocol involved combined amplification with forward and indexed reverse primers. After sequencing, the microbiome data were analyzed using Nephele (RRID: SCR_016595), a cloud‐based web application from the Office of Cyber Infrastructure and Computational Biology (OCICB), National Institute of Allergy and Infectious Diseases (http://nephele.niaid.nih.gov/; 2016). QIIME (RRID: SCR_008249) was used for analysis [26], and RStudio (RRID: SCR_000432) for statistical analysis [27]. Reads that were demultiplexed were clustered into OTUs using an open reference approach by comparison with the SILVA_99 database, allowing sequences to cluster at 99% similarity. Analyses included the identification of chimeras and removal using uchime. The abundance of each taxon was analyzed using the phyloseq package in RStudio [27]. The compositional heterogeneity of each sample's microbial community at every timepoint and each taxonomic level was visualized using PCoA scaling and the ordinate function in the phyloseq package and using the Bray–Curtis dissimilarity index calculated using the metaMDS function in the vegan package.

### 2.5 Whole‐Brain Proteomics

Whole brains were collected at the end of the experiment (day 28), frozen at −80°C, and shipped to the IDeA National Resource for Quantitative Proteomics, Little Rock, AR, for processing. Total protein from each sample was reduced, alkylated, and delipidated before digestion with sequencing‐grade modified porcine trypsin (Promega, Madison, WI). Tryptic peptides were then separated by a reverse phase Ion‐Opticks‐TS analytical column supported by an EASY‐Spray nano‐source and stabilized with a Heater THOR Controller (Ion‐Opticks, Fitzroy, Australia) at 60°C. Eluted peptides were ionized by electrospray (2.5 kV) and analyzed using an Orbitrap Astral mass spectrometer (Thermo Scientific). Data Analysis: Following data acquisition, data were searched using Spectronaut (Biognosys, version 20.1; Biognosys, Zurich, Switzerland) against the UniProt *Mus musculus* database (3rd version of 2025) using the directDIA method with an identification precursor and protein *q*‐value cutoff of 1%, generate decoys set to true, the protein inference workflow set to maxLFQ, inference algorithm set to IDPicker, quantity level set to MS2, cross‐run normalization set to false, and the protein grouping quantification set to median peptide and precursor quantity. Fixed Modifications were set to Carbamidomethyl (C), and variable modifications were set to Acetyl (Protein N‐term), Oxidation (M). Protein MS2 intensity values were assessed for quality using ProteiNorm [28]. Data normalization and statistical analysis are shown below.

### 2.6 Statistical Analysis

For EAE clinical scores and body weight changes, group differences were estimated using a mixed‐effects ANOVA with repeated measures and two factors (*time*, *treatment*), followed by Tukey's post hoc multiple comparisons, with *p* < 0.05 considered statistically significant. Group differences in disease onset and severity scores were evaluated using non‐parametric Kruskal‐Wallis followed by Dunn's multiple comparisons tests, with *p* < 0.05 considered statistically different. The R software package was used for the statistical analysis of microbiota and mycobiota data. ADONIS was used to determine group differences in gut microbiota and mycobiota compositions estimated by PCoA analysis (adjusted *p* values provided), and differences in microbiota alpha diversity and Firmicutes: Bacteroidetes (F/B) ratios were evaluated using non‐parametric Kruskal‐Wallis followed by Dunn's multiple comparisons testing, with *p* < 0.05 considered statistically different. A two‐way ANOVA complemented the statistical analysis of the microbiota and mycobiota using ADONIS with two independent variables, followed by Tukey's test to assess group differences at the phylum, family, and genus levels, yielding adjusted *p* values when comparing all mice and time points combined for each taxonomic level. The statistical analysis was performed with GraphPad Prism (RRID: SCR_002798), version 10. In the proteomics analysis, the data were normalized using Variance Stabilizing Normalization (VSN) [29] and analyzed using proteoDA to perform statistical analysis using Linear Models for Microarray Data (limma) with empirical Bayes (eBayes) smoothing to the standard errors [30, 31]. Proteins with an FDR‐adjusted *p* value < 0.05 and a fold change > 2 were considered significant.

## 3 Results

### 3.1 Treatment With GABA‐Producing P8s‐GAD *L. lactis* Protects Against EAE in Env Mice

**FIGURE 1:**
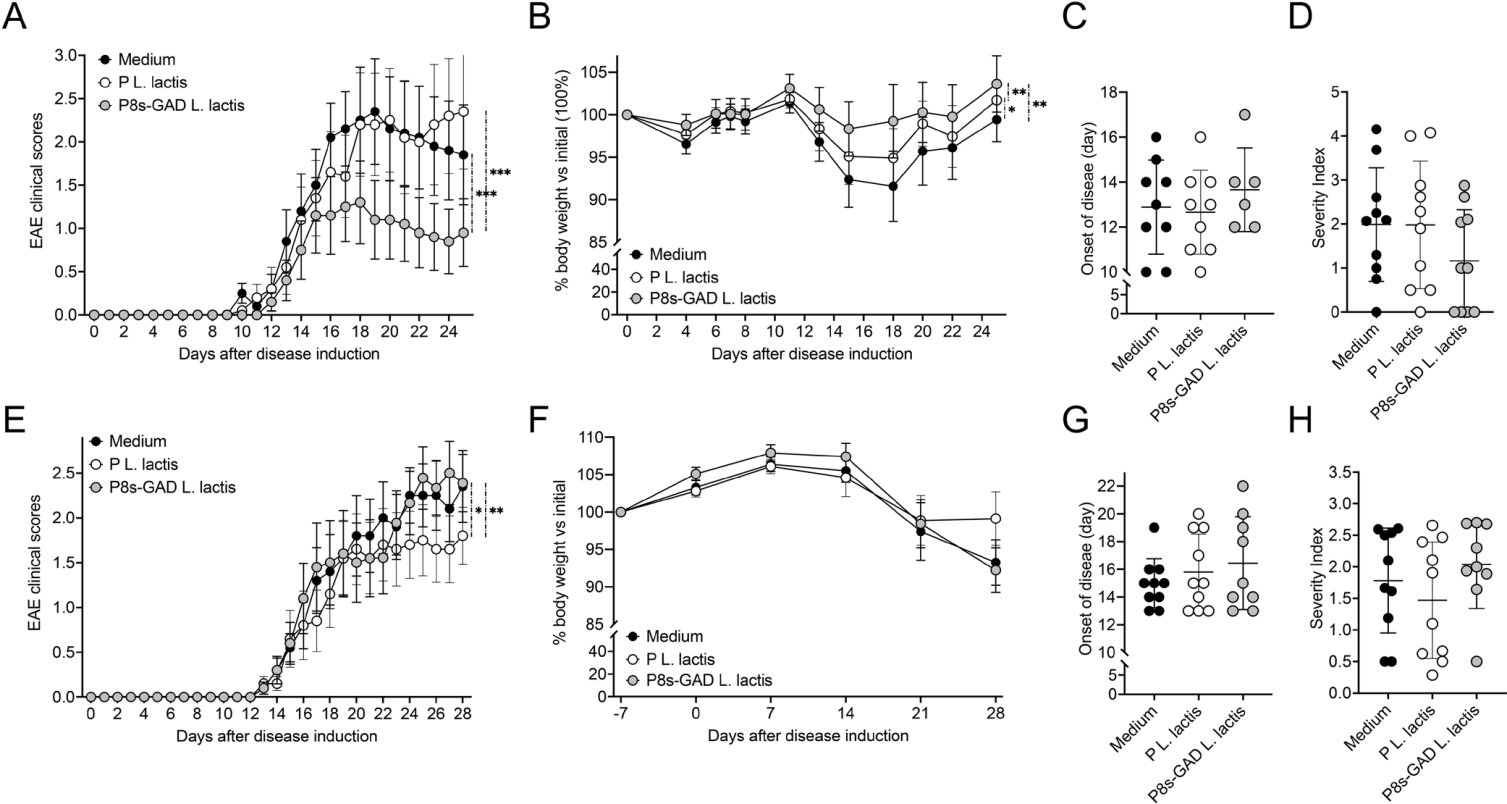
P8s‐GAD *L. lactis* treatment protects Env but not Jax C57BL/6 mice from EAE. (A–D) Env mice were treated by oral gavage with 100 μL of 5 × 10^8^ CFU (100 μL from 5 × 10^9^ CFU/mL cultures) of P8s‐GAD *L. lactis* or P‐ *L. lactis* , or 100 μL of GM17 medium (Medium), five consecutive days per week from the day of EAE induction (D0) until the end of the experiment. (A) Daily clinical scores represented as mean ± SEM, analyzed by repeated measures of ANOVA and Tukey's test; (B) Body weight percentage compared to the initial value at D0, represented as mean ± SEM, analyzed by repeated measures of ANOVA and Tukey's test; (C) EAE clinical onset depicted as the mean ± SEM, and compared by Mann–Whitney test. (D) EAE clinical severity, calculated as the cumulative scores divided by the number of days with EAE scores per mouse, depicted as the mean ± SEM, and compared by Mann–Whitney test. *p < 0.05; **p < 0.01. ***p < 0.001. *n* = 10 per group. (E–H) Jax mice were treated by oral gavage with 5 × 10^8^ CFU of P8s‐GAD *L. lactis* or P‐ *L. lactis* , or 100 μL of GM17 medium (Medium). Controls received GM17 medium without GA supplementation. Treatments were administered five consecutive days per week, beginning 7 days before EAE induction (D‐7) and continuing until the end of the experiment. (E) Daily clinical scores represented as mean ± SEM, analyzed by repeated measures of ANOVA and Tukey's test. (F) Body weight percentage compared to the initial value at D0, represented as mean ± SEM, analyzed by repeated measures of ANOVA and Tukey's test. (G) EAE clinical onset is depicted as the mean ± SEM and compared by the Mann–Whitney test. (H) EAE clinical severity, depicted as the mean ± SEM, and compared by Mann–Whitney test. *n* = 10 per group.

We first evaluated whether the oral treatment with P8s‐GAD *L. lactis* was protective against EAE. We used Env mice as previously done in other studies performed by our group [1, 2]. The treatments with GABA‐producing P8s‐GAD *L. lactis* , control (P‐ *L. lactis* ), and Medium (GM17 medium; Medium) were done five times per week (consecutive) and started at day 0 (day of EAE induction; Figure 1A–D). We observed a significant improvement in clinical scores in the P8s‐GAD *L. lactis* ‐treated group compared with the P‐ *L. lactis* group or the Medium group (Figure 1A). We also observed a significant and positive impact of P8s‐GAD *L. lactis* on body weight, with mice treated with P8s‐GAD *L. lactis* maintaining their pre‐induction body weights after induction and throughout the entire treatment period, whereas mice treated with either P‐ *L. lactis* or Medium showed the expected weight decline after EAE induction (Figure 1B). An increase in body weight averages was observed in the two control groups (P‐ *L. lactis* and Medium) toward the end of the treatment period. This apparent recovery is artifactual, as it reflects group attrition caused by euthanasia of the sickest mice, leaving only the less affected, hence heavier animals (two in Medium and three in P‐ *L. lactis* ; Figure 1C). No significant group differences in the onset of EAE disease were observed. However, a delayed trend was observed in mice subjected to P8s‐GAD *L. lactis* and P‐ *L. lactis* treatments compared to Medium mice (Figure 1D). When comparing the EAE severity index, no significant differences were observed between groups, but again, a trend was seen, suggesting delayed onset in the P8s‐GAD *L. lactis* and P‐ *L. lactis* when compared to Medium controls (Figure 1D). An analysis of the distribution of the EAE scores shows that the P8s‐GAD *L. lactis group* had a higher number of mice with EAE scores of 0 or 1 at the approximate peak of the disease (day 19) and at the end of the experiment (day 25; Figure S1A). These results indicate that the oral administration of P8s‐GAD *L. lactis*, starting at the time of disease induction, has a significant impact on the progression of EAE induced in Env mice, despite no effects observed on when disease is first detected.

In contrast to what we observed in Env mice, GABA‐producing P8s‐GAD *L. lactis* conferred no significant clinical protection in Jax mice. We had previously shown that the microbiota composition of naive and EAE Jax mice differed from that of C57BL/6 Env [2]. This earlier finding raised the possibility that the absence of protection was attributable to differences in the gut microbiota prior to induction and to differences in the gut microbiota response to EAE induction. We first tested if oral administration started at EAE induction (Day 0) would result in protection against disease as observed in Env mice (Figure S1B–F). An improvement of the clinical scores of mice treated with P8s‐GAD *L. lactis* compared to P‐ *L. lactis* was observed when the experiment was terminated at the peak of the disease. However, there was no difference in clinical score between P8s‐GAD *L. lactis* and the Medium control group, and no differences in body weight, onset of disease, or severity index (cumulative scores divided by the number of days with active EAE scores) were observed. Score distributions were not significantly different.

We next tested whether a longer treatment (started at Day −7) would result in protection against EAE (Figure 1E–H). The treatment with P‐ *L. lactis* in this experiment reduced EAE clinical score averages compared to Medium‐ and P8s‐GAD *L. lactis* ‐treated Jax mice (Figure 1E). Again, we observed no differences in body weight (Figure 1F), the onset of disease (Figure 1G), or severity index (Figure 1H). We also tested whether constant exposure to the probiotics through drinking water administration would protect against EAE (Figure S1G–K). In this experiment, probiotic administration began on day −7 (7 days prior to EAE induction) and continued for 28 days. Administration of P8s‐GAD *L. lactis* significantly reduced disease by day 21, which is the peak of disease in the control (Medium‐treated) group, based on our previous observations, compared with P‐ *L. lactis* and Medium controls (Figure S1H). However, no differences in body weight, disease onset, or severity index were observed (Figure S1I–K).

Together, these studies show that the GABA‐producing P8s‐GAD *L. lactis* strain does not confer protection against EAE in Jax mice, in contrast to our observations in Env mice.

### 3.2 P8s‐GAD *L. lactis* and P‐ *L. lactis* Effects on Gut Microbiota

**FIGURE 2:**
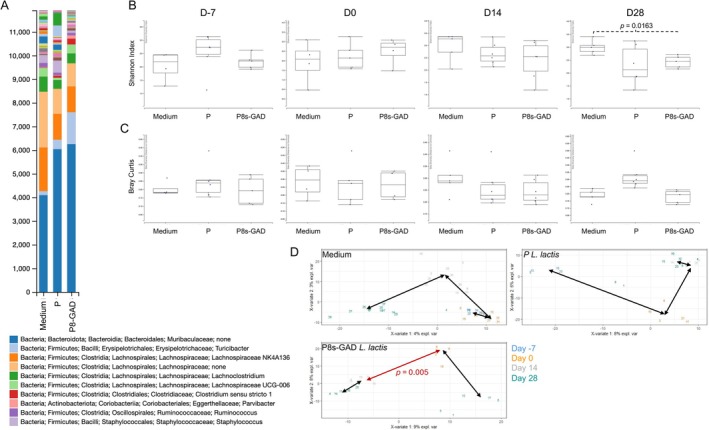
P8s‐GAD *L. lactis* treatment modifies gut microbiome in Jax mice. Mice were treated by oral gavage with 100 μL of 5 × 10^8^ CFU of P8s‐GAD *L. lactis* or P‐ *L. lactis* , or 100 μL of GM17 medium (Medium), five consecutive days per week, starting 7 days before EAE induction (D‐7) until the end of the experiment. Stool samples were collected on D‐7, D0, D14, and D28. (A) Bar plot represents the relative abundances of the gut microbiome at the genus level on D28; (B) Alpha diversity (Genus level) measured by Shannon index on D‐7, D0, D14, and D28. Non‐parametric Wilcoxon‐Sum Rank; (C) Bray‐Curtis beta diversity quantified by distance to the centroid on D‐7, D0, D14, and D28. Permanova analysis (Number of permutations: 999); (D) Beta diversity within timepoints for each treatment group. Visualization by two‐dimensional Plot of Individuals (PlotInd) with pairs of groups (timepoints) analyzed with Adonis. *p values < 0.05 are considered significant.

We sought to further investigate the origin of such vendor differences and postulated that the gut microbiota response to P8s‐GAD *L. lactis* administration may have offset the clinical benefit of P8s‐GAD *L. lactis* treatment. To test this hypothesis, we studied the gut microbiota composition in EAE‐induced Jax mice treated with P8s‐GAD *L. lactis* (oral gavage) from day −7 until Day 28 (Figure 1E–H). Samples were obtained on days −7, 0 (day of EAE induction), 14, and 28 (end of experiment). We first compared the microbiome taxonomical composition in the three treatment groups (GM17: Medium; P‐ *L. lactis* : P; and P8s‐GAD *L. lactis* : P8s) at the genus level, on D‐7, D0, D14 (Figure S3), and D28 (Figure 2A). The composition bar plots illustrate the shift in microbiome composition over time and the overall differences between groups. At the end of the experiment (D28), increases in unidentified genera of the Families *Moribaculaceae* (Bacteroidia) and *Lachnospiraceae* (Clostridia) were observed. Samples from P8s‐GAD *L. lactis* ‐treated mice had a reduction in alpha diversity (Shannon Index) compared to Medium‐treated mice by the end of the experiment, while no differences were observed with P‐ *L. lactis* (D28) (Figure 2B). No differences were observed in the alpha diversity at earlier timepoints and between samples of Medium‐ and P‐ *L. lactis* ‐treated mice (Figure 2B). A similar time‐dependent pattern was observed with beta diversity (Bray–Curtis distance to centroid after 999 permutations). However, there were no statistically significant differences between groups (Figure 2C). Only samples obtained from P8s‐GAD *L. lactis* ‐treated mice showed differences in beta diversity between experimental times (D14 vs. D28), suggesting a synergistic effect of EAE progression and treatment with the GABA‐producing probiotic, where disease and the probiotic treatment exacerbate microbial modifications, as shown in the beta diversity analysis and resulting *p* values in Medium‐treated and P8s‐GAD *L. lactis* ‐treated mice plots (Figure 2D).

We next compared the differential abundances at the phylum taxonomic level. No significant differences were found between groups at timepoints D‐7, D0, and D14 (not shown). At the end of the experiment (D28), compared to Medium‐treated EAE mice, the samples from mice treated with P8s‐GAD *L. lactis* had a significant increase in the relative abundance of members of the phylum Bacteroidota (*p* = 0.0282) and Actinobacteria (*p* = 0.0282), and a significant reduction in the phylum Firmicutes (*p* = 0.0282; Figure S2). Actinobacteria were also significantly increased in P8s‐GAD *L. lactis* compared to P‐ *L. lactis* ‐treated mice (*p* = 0.0446; Figure S2). Comparing the firmicutes: bacteroidetes (F:B) ratio, no statistical differences were found compared to Medium mice. No changes were observed in mice treated with P‐ *L. lactis* compared to controls or P8s‐GAD *L. lactis* (Figure S2).

Next, we compared the differential abundances at the Family taxonomic level (Table 1). No significant differences were found between groups at the beginning of the experiment (D‐7). On D0, after 7 days of treatments, the relative abundances of an unknown family of Clostridia vadinBB60 group (*p* = 0.0209), an unknown family of Clostridia UCG‐014 (*p* = 0.0209), and *Oscillospiraceae* (*p* = 0.0443) were significantly increased in P8s‐GAD *L. lactis* compared to Medium‐treated mice (Figure S4A). Samples from P‐ *L. lactis* ‐treated mice had increased relative abundances of *Bacteroidaceae* compared to P8s‐GAD *L. lactis* ‐treated mice (*p* = 0.0143). Compared to Medium, P‐ *L. lactis* ‐treated mice had a significantly increased abundance of Oscillospiraceae (*p* = 0.0274) and a reduction in *Ruminococcaceae* (*p* = 0.0274; Figure S4A). On D14, *Bacteroidaceae* were significantly increased in P‐ *L. lactis* ‐treated mice compared to Medium (*p* = 0.0248) and to P8s‐GAD *L. lactis* ‐treated mice (*p* = 0.0163; Figure S4B). Most of the differences in the relative abundances within the family taxa level were observed at the end of the experiment (D28) (Figure S4C). Compared to Medium, the abundances of *Muribaculaceae* (*p* = 0.0282), *Eggerthellaceae* (*p* = 0.0282), and *Peptostreptococcaceae* (*p* = 0.0367) were significantly increased in samples from P8s‐GAD *L. lactis* ‐treated mice. By contrast, *Lachnospiraceae* (*p* = 0.0162), *Lactobacillaceae* (*p* = 0.0472), *Oscillospiraceae* (*p* = 0.0472), and *Bacteroidaceae* (*p* = 0.009) were significantly reduced in P8s‐GAD *L. lactis* ‐treated mice compared to samples from Medium‐treated EAE mice (Figure S4C). *Lachnospiraceae* were significantly reduced in P‐ *L. lactis* ‐treated mice compared to Medium mice (*p* = 0.0446). Compared to P8s‐GAD *L. lactis* ‐treated mice, *Bacteroidaceae* was significantly increased in P‐ *L. lactis* ‐treated mice (*p* = 0.0224), while *Eggerthellaceae* (*p* = 0.0284) and *Peptostreptococcaceae* (*p* = 0.0284) were significantly reduced (Figure S4C).

**TABLE 1:** Bacterial family‐level differential abundances between treatments observed during EAE.^a^

	Family	*p*	*Q*‐value
Day −7 (D‐7)			
Medium vs. P	—	—	—
Medium vs. P8s	—	—	—
P vs. P8s	—	—	—
Day 0 (D0)			
Medium vs. P	*Oscillospiraceae*	0.0275	0.2199
	*Ruminococcaceae*	0.0275	0.2199
Medium vs. P8s	*Clostridia vadinBB60 group; none*	0.0209	0.1673
	*Clostridia UCG‐014; none*	0.0209	0.1673
	*Oscillospiraceae*	0.0433	0.2309
P vs. P8s	*Bacteroidaceae*	0.0143	0.2289
Day 14 (D14)			
Medium vs. P	—	—	—
Medium vs. P8s	*Bacteroidaceae*	0.0284	0.4214
P vs. P8s	*Bacteroidaceae*	0.0163	0.3425
Day 28 (D28)			
Medium vs. P	*Lachnospiraceae*	0.0446	0.6897
Medium vs. P8s	*Muribaculaceae*	0.0282	0.1416
	*Lachnospiraceae*	0.0163	0.1416
	*Lactobacillaceae*	0.0472	0.1416
	*Oscillospiraceae*	0.0472	0.1416
	*Bacteroidaceae*	0.0090	0.1416
	*Eggerthellaceae*	0.0282	0.1416
	*Peptostreptococcaceae*	0.0367	0.1416
P vs. P8s	*Bacteroidaceae*	0.0224	0.1992
	*Eggerthellaceae*	0.0284	0.1992
	*Peptostreptococcaceae*	0.0284	0.1992

^a^ Family level. Wilcoxon Sum‐Rank analysis D‐7: day −7 (7 days prior EAE induction; when treatments started); D0: day 0 (day of EAE induction); D14: day 14 after EAE induction; D28: day 28 after EAE induction.

A Wilcoxon Rank‐Sum analysis was performed on data sets from the three groups at each timepoint at the genus level, providing significant differences at *p* values < 0.05. *Q* values for each comparison are shown in Table 2. On D‐7, the microbiome composition was mostly unaltered, with only an increased relative abundance of A2 of *Lachnospiraceae* in the P‐ *L. lactis* group compared to Medium (*p* = 0.0428) and P8s‐GAD *L. lactis* (*p* = 0.042; Figure S5A). After one week of treatments and right before EAE induction (D0), no differences in relative abundances of genera were observed between Medium‐ and P‐L. lactis‐treated mice (including A2 of *Lachnospiraceae*), and Medium‐ and P8s‐GAD *L. lactis‐treated* mice. However, a significant increase in *Bacteroides* was observed in P‐ *L. lactis* compared to P8s‐GAD *L. lactis* ‐treated mice (Figure S5B). On D14, the genus *Marvinbryantia* members' relative abundance was increased in P‐ *L. lactis* compared to Medium (*p* = 0.04460) and P8s‐GAD *L. lactis* ‐treated mice (*p* = 0.0249; Figure S5C). *Oscillibacter* (*p* = 0.0284) and *Bacteroides* (*p* = 0.0284) were significantly reduced in P8s‐GAD L. *lactis* when compared to Medium (*p* = 0.0284). *Bacteroides* were also significantly increased in P‐ *L. lactis* compared to P8s‐GAD *L. lactis* (*p* = 0.0163; Figure S5C). At the end of the experiment (D28), *Lachnospiraceae* UCG‐006 (*p* = 0.04460) and *Lachnospiraceae* UCG‐004 (*p* = 0.0284) were significantly increased in Medium mice compared to P‐ *L. lactis* , but not to P8s‐GAD *L. lactis* (Figure S5D). *Lachnospiraceae* UCG‐006 was further reduced in P8s‐GAD *L. lactis* compared to P‐ *L. lactis* (*p* = 0.04460; Figure S5C). *Bacteroides* were significantly reduced in P8s‐GAD *L. lactis* compared to Medium (*p* = 0.009) and P‐ *L. lactis* (*p* = 0.0224). *Marvinbryantia* (*p* = 0.0162) and Oscillibacter (*p* = 0.0282) were reduced in P8s‐GAD *L. lactis* compared to Medium‐treated mice. By contrast, the relative abundance of the genus Romboutsia significantly increased in P8s‐GAD *L. lactis* compared to Medium (*p* = 0.0367) and P‐ *L. lactis* (*p* = 0.0284). Parvibacter also showed a significant increase in P8s‐GAD *L. lactis* compared to P‐ *L. lactis* (*p* = 0.0446), but not to Medium‐treated mice (Figure S5D).

**TABLE 2:** Bacterial genus‐level differential abundances between treatments observed during EAE.^a^

	Genus	*p*	*Q*‐value
Day −7 (D‐7)			
Medium vs. P	*Lachnospiraceae*; A2	0.0428	0.6899
Medium vs. P8s	—	—	—
P vs. P8s	*Lachnospiraceae*; A2	0.042	0.9458
Day 0 (D0)			
Medium vs. P	—	—	—
Medium vs. P8s	*Clostridia vadinBB60 group; none*	0.0209	0.2650
	*Clostridia UCG‐014; none*	0.0209	0.2650
	*Oscillospiraceae; none*	0.0209	0.2650
P vs. P8s	*Bacteroides*	0.0143	0.5436
Day 14 (D14)			
Medium vs. P	*Marvinbryantia*	0.0446	0.9230
Medium vs. P8s	*Oscillibacter*	0.0284	0.6688
	*Bacteroides*	0.0284	0.6688
P vs. P8s	*Marvinbryantia*	0.0249	0.5869
	*Bacteroides*	0.0163	0.5869
Day 28 (D28)			
Medium vs. P	*Lachnospiraceae UCG‐006*	0.0446	0.6529
	*Lachnospiraceae UCG‐004*	0.0284	0.6529
Medium vs. P8s	*Lachnospiraceae; none*	0.0090	0.1940
	*Bacteroides*	0.0090	0.1940
	*Marvinbryantia*	0.0162	0.2335
	*Muribaculaceae; none*	0.0282	0.2432
	*Oscillibacter*	0.0282	0.2432
	*Romboutsia*	0.0367	0.2631
	*Oscillospiraceae; none*	0.0472	0.2899
P vs. P8s	*Lachnospiraceae UCG‐006*	0.0446	0.4795
	*Bacteroides*	0.0224	0.4795
	*Parvibacter*	0.0446	0.4795
	*Romboutsia*	0.0284	0.4795

^a^ Genus level. Wilcoxon Sum‐Rank analysis D‐7: day −7 (7 days prior EAE induction; when treatments started); D0: day 0 (day of EAE induction); D14: day 14 after EAE induction; D28: day 28 after EAE induction.

### 3.3 P8s‐GAD *L. lactis* and P‐ *L. lactis* Effects on Gut Mycobiota

**FIGURE 3:**
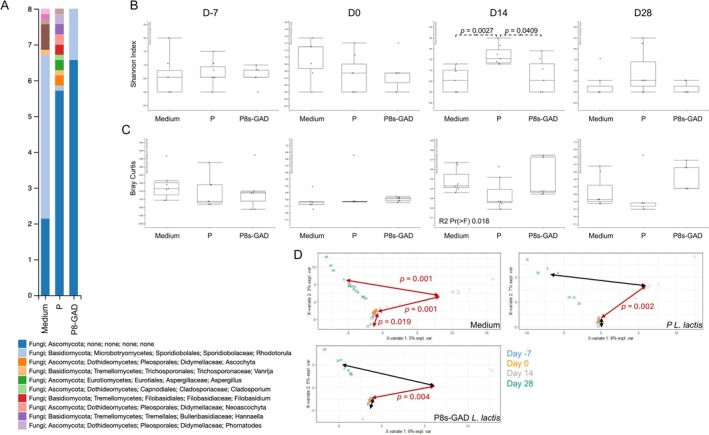
P8s‐GAD *L. lactis* treatment modifies gut mycobiome in Jax mice. Mice were treated by oral gavage with 100 μL of 5 × 10^8^ CFU of P8s‐GAD *L. lactis* or P‐ *L. lactis* , or 100 μL of GM17 medium (Medium), five consecutive days per week, starting seven days before EAE induction (D‐7) until the end of the experiment, as depicted in the graphical representation in Figure 2C. (A) Barplot represents the relative abundances of the gut mycobiome at the genus level on D28; (B) Alpha diversity (Genus level) measured by Shannon index on D‐7, D0, D14, and D28. Non‐parametric Wilcoxon‐Sum Rank; (C) Bray‐Curtis beta diversity quantified by distance to the centroid on D‐7, D0, D14, and D28. Permanova analysis (Number of permutations: 999); (D) Beta diversity within timepoints for each treatment group. Visualization by two‐dimensional Plot of Individuals (PlotInd) with pairs of groups (timepoints) analyzed with Adonis. *p values < 0.05 are considered significant.

We next analyzed whether the treatment of EAE with probiotics would impact the fungal compartment of the gut microbial composition (the mycobiome). As done before, when comparing the microbiota among groups and timepoints, we first performed a barplot analysis on D‐7, D0, D14, and D28. The results indicate that the mycobiota shifted over time, mainly in the mice treated with the Medium on D‐7, D0, and D14 (Figure S6) and D28 (Figure 3). Comparing alpha diversity, only P‐ *L. lactis* vs. P8s‐GAD *L. lactis* and P‐ *L. lactis* vs. Medium on D14 showed statistical differences (increased in P‐ *L. lactis* ; Figure 3A). Bray–Curtis distance to centroid analysis of beta diversity showed a significant difference on D14 (Figure 3B). Interestingly, time course analysis of beta diversity showed that EAE resulted in significant differences over time (D‐7 vs. D0, D0 vs. D14, and D14 vs. D28). A significant difference was observed in P‐ *L. lactis* or P8s‐GAD *L. lactis* at the early stages of EAE (D0 vs. D14) but not later (Figure 3C).

We compared the samples using heatmap analysis across all time points. The samples from P8s‐GAD *L. lactis* ‐treated mice showed the most diverse profile on D14 compared to Medium‐treated mice, while being similar to P‐ *L. lactis* ‐treated mice (Figure S7A). By D28, the profile showed a reduced diversity in abundances in all groups. The reduction in overall diversity was more accentuated in medium‐treated EAE mice than in P‐*L*. *lactis*‐ and P8s‐GAD‐ *L. lactis* ‐treated mice. The profiles for D‐7 and D0 were similar for all groups (Figure S7A). We assessed whether the abundances of an unknown Ascimycota genus, most abundant as shown in the barplot (Figure S7B), showed no significant differences. Similarly, when the differential abundances across all taxonomic levels were compared, no significant effects were observed in any group comparisons (not shown). Our results, albeit with small group sizes, suggest that the effects of *L. lactis* probiotics were more pronounced in the bacterial than in the fungal gut compartment.

### 3.4 Supplementation With Glutamic Acid Is Not Sufficient to Improve the Protective Effects of GABA‐Producing P8s‐GAD *L. lactis* in Jax Mice

**FIGURE 4:**
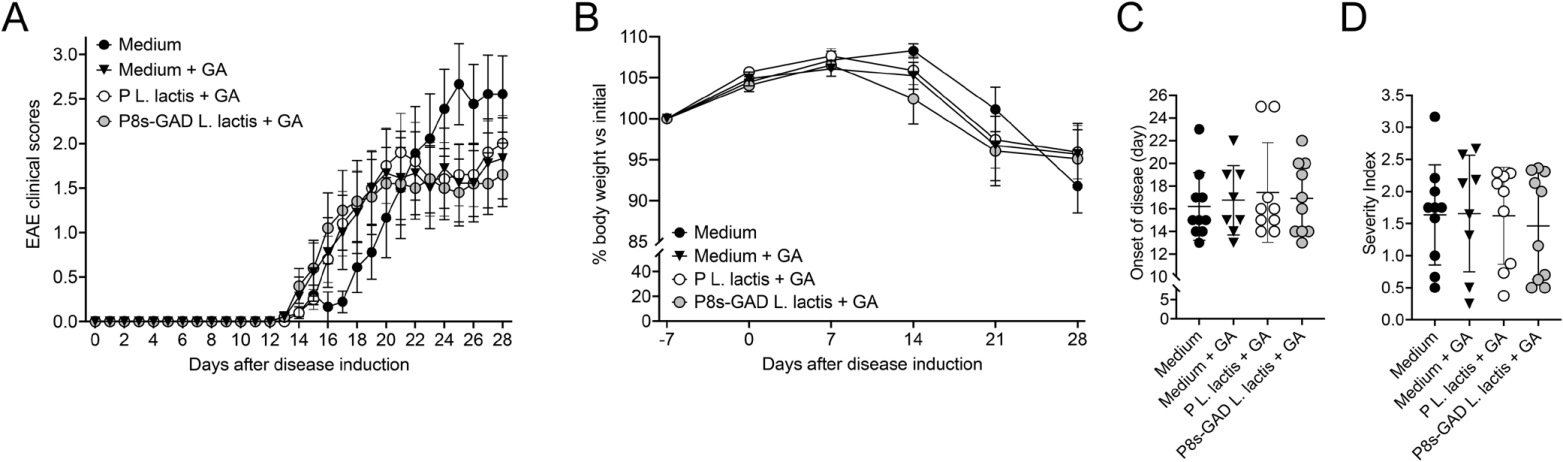
Co‐administration of GA does not improve the protective effects of P8s‐GAD *L. lactis* against EAE in Jax mice. Jax mice were treated by oral gavage with 5 × 10^8^ CFU of P8s‐GAD *L. lactis* or P‐ *L. lactis* , or 100 μL of GM17 medium (Medium), supplemented with 200 mM glutamic acid hydrochloride (GA). Controls received GM17 medium without GA supplementation. Treatments were administered five consecutive days per week, beginning 7 days before EAE induction (D‐7) and continuing until the end of the experiment. (A) Daily clinical scores represented as mean ± SEM, analyzed by repeated measures of ANOVA and Tukey's test. (B) Body weight percentage compared to the initial value at D0, represented as mean ± SEM, analyzed by repeated measures of ANOVA and Tukey's test. (C) EAE clinical onset is depicted as the mean ± SEM and compared by the Mann–Whitney test. (D) EAE clinical severity, depicted as the mean ± SEM, and compared by Mann–Whitney test. *n* = 10 per group.

Since GABA production by P8s‐GAD *L. lactis* increases when cultured in 200 mM glutamic acid hydrochloride (GA) [20], we assessed whether the administration of the bacterium supplemented with GA at that concentration would improve the efficacy of the treatment with the bacterium (Figure 4). The treatment regime and doses were the same as shown in Figure 1E–H, with the addition of 200 mM of GA to Medium, P‐ *L. lactis* , and P8s‐GAD *L. lactis* . A Medium control group, not receiving GA, was also included. No significant differences were observed in the EAE clinical scores (Figure 4A), body weights (Figure 4B), disease onset (Figure 4C), and disease severity (Figure 4D). Since the M17 medium used to formulate GM17 already contains 0.66 mM of glutamic acid [32], we also tested whether GA alone (dissolved in drinking water) would impact the progression of EAE (Figure S8). The addition of 200 mM GA to water did not affect how the disease progressed in mice (Figure S8).

We compared the effects of P‐ *L. lactis* and P8s‐GAD *L. lactis* administration supplemented with 200 mM GA on the microbiome and mycobiome composition of EAE mice with those treated with medium supplemented with GA. First, we studied the impact of GA supplementation on the composition of the microbiome and mycobiome by comparing EAE mice treated with 200 mM GA administered in Medium or Medium without GA (Figure S9). No significant effects were observed in the alpha diversity by 16S rDNA sequencing (Figure S9A) and by ITS sequencing (Figure S9B). Beta diversity, visualized by two‐dimensional Plot of Individuals (PlotInd) with pairs of groups (all timepoints) analyzed with Adonis showed no differences in the microbiome compartment (Figure S9C), and only a significant difference between D0 and D14 in the mycobiome (Figure S9D). The taxonomic profiles of GA treated and untreated EAE mice for each timepoint are shown in Figures S10 and S11, for the microbiome and mycobiome, respectively. Despite the D0‐D14 difference in the mycobiome beta diversity, a non‐parametric Wilcoxon Rank‐Sum analysis comparing both groups at each timepoint did not report any taxa significantly altered. These results suggest that GA's impact on the microbiome and mycobiome, alone, at the concentration administered, is minimal.

**FIGURE 5:**
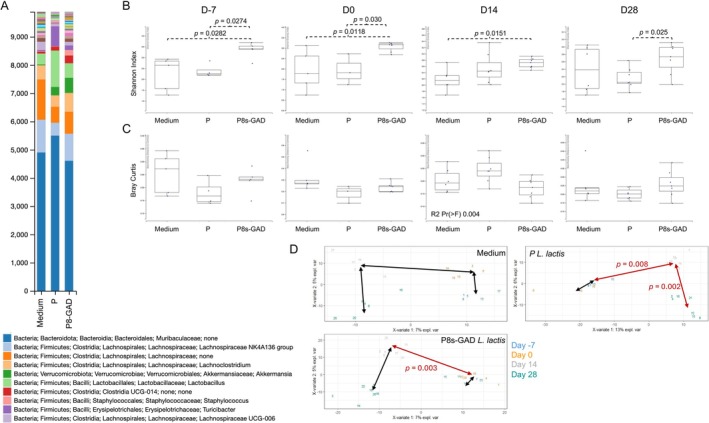
Co‐administration of GA and P8s‐GAD *L. lactis* modifies gut microbiome in Jax mice. Mice were treated by oral gavage with 100 μL of 5 × 10^8^ CFU of P8s‐GAD *L. lactis* or P‐ *L. lactis* , or 100 μL of GM17 medium (Medium), co‐administered with 200 mM GA, five consecutive days per week, starting 7 days before EAE induction (D‐7) until the end of the experiment. Stool samples were collected on D‐7, D0, D14, and D28. (A) Bar plot represents the relative abundances of the gut microbiome at the genus level on D28; (B) Alpha diversity (Genus level) measured by Shannon index on D‐7, D0, D14, and D28. Non‐parametric Wilcoxon‐Sum Rank; (C) Bray‐Curtis beta diversity quantified by distance to the centroid on D‐7, D0, D14, and D28. Permanova analysis (Number of permutations: 999); (D) Beta diversity within timepoints for each treatment group. Visualization by two‐dimensional Plot of Individuals (PlotInd) with pairs of groups (timepoints) analyzed with Adonis. *p values < 0.05 are considered significant.

We compared the taxonomic composition of the microbiome in the three treatment groups (GM17 + GA, Medium+GA, and P‐ *L. lactis* + GA; P8s‐GAD *L. lactis* + GA) supplemented with 200 mM GA at the genus level on D‐7, D0, D14 (Figure S12), and D28 (Figure 5A). P8s‐GAD‐ *L. lactis* + GA showed an increase in alpha diversity compared to medium+GA and P‐ *L. lactis* ‐treated+GA mice on D‐7, D0, and D14, and to P‐ *L. lactis* ‐treated mice on D28 (Figure 5B). Beta diversity analysis by Bray‐Curtis distance to centroid showed that D14 offered the most distinct profiles (Figure 5C), confirmed by two‐dimensional Plot of Individuals (PlotInd), showing differences between D0 and D14 for P‐ *L. lactis* ‐treated+GA and P8s‐GAD‐ *L. lactis* + GA. D14‐D28 was also significant in P‐ *L. lactis* ‐treated+GA. At the phylum level, the addition of GA to the treatments caused no significant impact on the abundances or F:B ratio (Figure S13). Overall, the effects at the Family and Genus levels were more modest than those observed without the addition of GA. We performed Wilcoxon Rank‐Sum analysis on data sets from the three groups at each timepoint, at the family (Table 3) and genus (Table 4) levels. Significant differences in abundance were observed at D0 and D14, but not at the end of the experiment (D28). On D0, *Clostridia. uncultured cluster group (UCG) 014;none* (Fam. *Lachnospiraceae*; Medium+GA vs. P‐ *L. lactis* + GA), Clostridia.UCG.014; none and Akkermansiaceae (Medium+GA vs. P8s‐GAD‐‐ *L. lactis* + GA, and P‐ *L. lactis* vs. P8s‐GAD‐‐ *L. lactis* + GA) were significantly different (Table 3). Akkermansiaceae was significantly overexpressed in P8s‐GAD‐‐ *L. lactis* + GA compared to P‐ *L. lactis* , while Clostridia.UCG.014; none of the abundances were reduced in P8s‐GAD‐‐ *L. lactis* + GA compared to P‐ *L. lactis* and with Medium+GA (Figure S14A). On D14, only the abundances of *Akkermansiaceae* were significantly different, comparing Medium+GA with P8s‐GAD‐ *L. lactis* + GA and P8s‐GAD‐‐ *L. lactis* + GA compared to P‐ *L. lactis* (Table 3), being overabundant in P8s‐GAD‐ *L. lactis* + GA (Figure S14B). At the genus level, D0, D14, and D28 showed significant differences between P8s‐GAD‐ *L. lactis* + GA and Medium+GA, and between P8s‐GAD‐ *L. lactis* + GA and P‐ *L. lactis* , but not between Medium+GA and P‐ *L. lactis* (Table 4). At D0, *Clostridia UCG‐014, none*, and *Eubacterium siraeum* .*group* were significantly different between P8s‐GAD‐ *L. lactis* + GA and Medium+GA, while *Akkermansia* was significantly different between P8s‐GAD‐ *L. lactis* + GA and P‐ *L. lactis* (Table 4). Group comparisons showed significant increases of *Akkermansia* and a reduction in *Clostridia:UCG‐014;none* in P8s‐GAD‐ *L. lactis* + GA compared to Medium+GA and P‐ *L. lactis* on D0 (Figure S14C), and increased abundance of *Akkermansia* in P8s‐GAD‐ *L. lactis* + GA compared to Medium+GA and P‐ *L. lactis* on D14 (Figure S14D).

**TABLE 3:** Bacterial family‐level differential abundances between treatments observed during EAE in mice supplemented with GA.^a^

	Genus	*p*	*Q*‐value
Day −7 (D‐7)			
Medium vs. P	—	—	—
Medium vs. P8s	—	—	—
P vs. P8s	—	—	—
Day 0 (D0)			
Medium vs. P	*Lachnospiraceae—Clostridia.UCG.014; none*	0.0178	0.2678
Medium vs. P8s	*Lachnospiraceae—Clostridia.UCG.014; none*	0.0012	0.0189
	*Akkermansiaceae*	0.0101	0.0757
P vs. P8s	*Akkermansiaceae*	0.0083	0.125
Day 14 (D14)			
Medium vs. P	—	—	—
Medium vs. P8s	*Akkermansiaceae*	0.0209	0.3566
P vs. P8s	*Akkermansiaceae*	0.0209	0.3566
Day 28 (D28)			
Medium vs. P	—	—	—
Medium vs. P8s	—	—	—
P vs. P8s	—	—	—

^a^ Genus level. Wilcoxon Sum‐Rank analysis D‐7: day −7 (7 days prior EAE induction; when treatments started); D0: day 0 (day of EAE induction); D14: day 14 after EAE induction; D28: day 28 after EAE induction.

**TABLE 4:** Bacterial genus‐level differential abundances between treatments observed during EAE in mice supplemented with GA.^a^

	Genus	*p*	*Q*‐value
Day −7 (D‐7)			
Medium vs. P	—	—	—
Medium vs. P8s	—	—	—
P vs. P8s	—	—	—
Day 0 (D0)			
Medium vs. P	—	—	—
Medium vs. P8s	*Clostridia UCG‐014;none;none*	0.02857	0.5143
	*Eubacterium siraeum* .*group*	0.02857	0.5143
P vs. P8s	*Akkermansia*	0.02857	1
Day 14 (D14)			
Medium vs. P	—	—	—
Medium vs. P8s	*Eubacterium siraeum* .*group*	0.04762	1
P vs. P8s	*Akkermansia*	0.04762	1
	*Lachnospiraceae;28.4*	0.04762	1
Day 28 (D28)			
Medium vs. P	—	—	—
Medium vs. P8s	*Lachnospiraceae UCG‐001*	0.04762	0.95238
	*RF39;none;none*	0.04762	1
P vs. P8s	*Lachnospiraceae UCG‐001*	0.04762	1

^a^ Genus level. Wilcoxon Sum‐Rank analysis D‐7: day −7 (7 days prior EAE induction; when treatments started); D0: day 0 (day of EAE induction); D14: day 14 after EAE induction; D28: day 28 after EAE induction.

**FIGURE 6:**
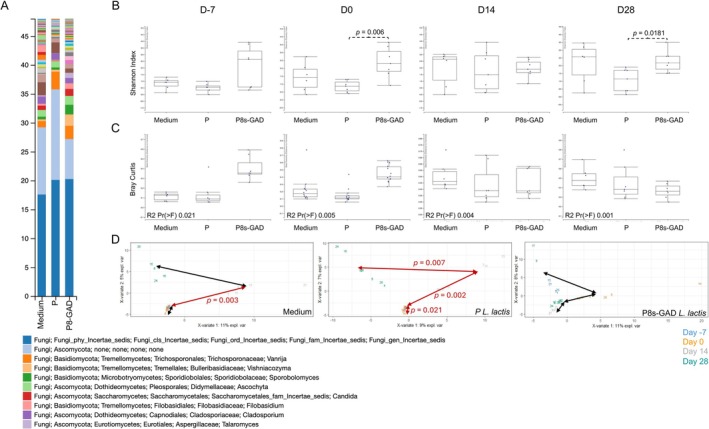
Co‐administration of GA and P8s‐GAD *L. lactis* modifies gut mycobiome in Jax mice. Mice were treated by oral gavage with 100 μL of 5 × 10^8^ CFU of P8s‐GAD *L. lactis* or P‐ *L. lactis* , or 100 μL of GM17 medium (Medium), co‐administered with 200 mM GA, five consecutive days per week, starting 7 days before EAE induction (D‐7) until the end of the experiment, as depicted in the graphical representation in Figure 2C. (A) Barplot represents the relative abundances of the gut mycobiome at the genus level on D28; (B) Alpha diversity (Genus level) measured by Shannon index on D‐7, D0, D14, and D28. Non‐parametric Wilcoxon‐Sum Rank; (C) Bray‐Curtis beta diversity quantified by distance to the centroid on D‐7, D0, D14, and D28. Permanova analysis (Number of permutations: 999); (D) Beta diversity within timepoints for each treatment group. Visualization by a two‐dimensional Plot of Individuals (PlotInd) with pairs of groups (timepoints) analyzed with Adonis. *p values < 0.05 are considered significant.

We compared the mycobiome composition of the treatment groups (GM17: Medium+GA, P‐ *L. lactis* + GA, and P8s‐GAD *L. lactis* + GA) supplemented with 200 mM GA. The relative abundance profiles for D‐7, D0, and D14 are shown in Figure S15, and at the end of the experiment, D28, in Figure 6A. The analysis showed an increase in the alpha diversity in P8s‐GAD *L. lactis* + GA versus P‐ *L. lactis* + GA, but no differences with Medium+GA (Figure 6B). Beta diversity differences were observed among groups at all timepoints. However, increased values were observed in P8s‐GAD *L. lactis* + GA compared to other groups (Figure 6C). Overall, beta diversity by two‐dimensional Plot of Individuals (PlotInd) showed significant differences between D0 and D14 in Medium+GA‐treated mice, and between D‐7 and D0, D0 and D14, and D14 and D28 in P‐ *L. lactis* + GA‐treated mice (Figure 6D). Surprisingly, no differences in beta diversity in mice treated with P8s‐GAD *L. lactis* + GA were observed (Figure 6D). Despite the lack of significance in beta diversity, the taxonomical analysis reported a significant increase in the relative abundance of an unknown taxa (*Incertae sedis*), at family, genus, and species level in P8s‐GAD *L. lactis* + GA compared to P‐ *L. lactis* + GA and Medium+GA (Figure S16).

### 3.5 Treatment With the *L. lactis* Strains Alters the Brain Proteome of EAE Mice

**FIGURE 7:**
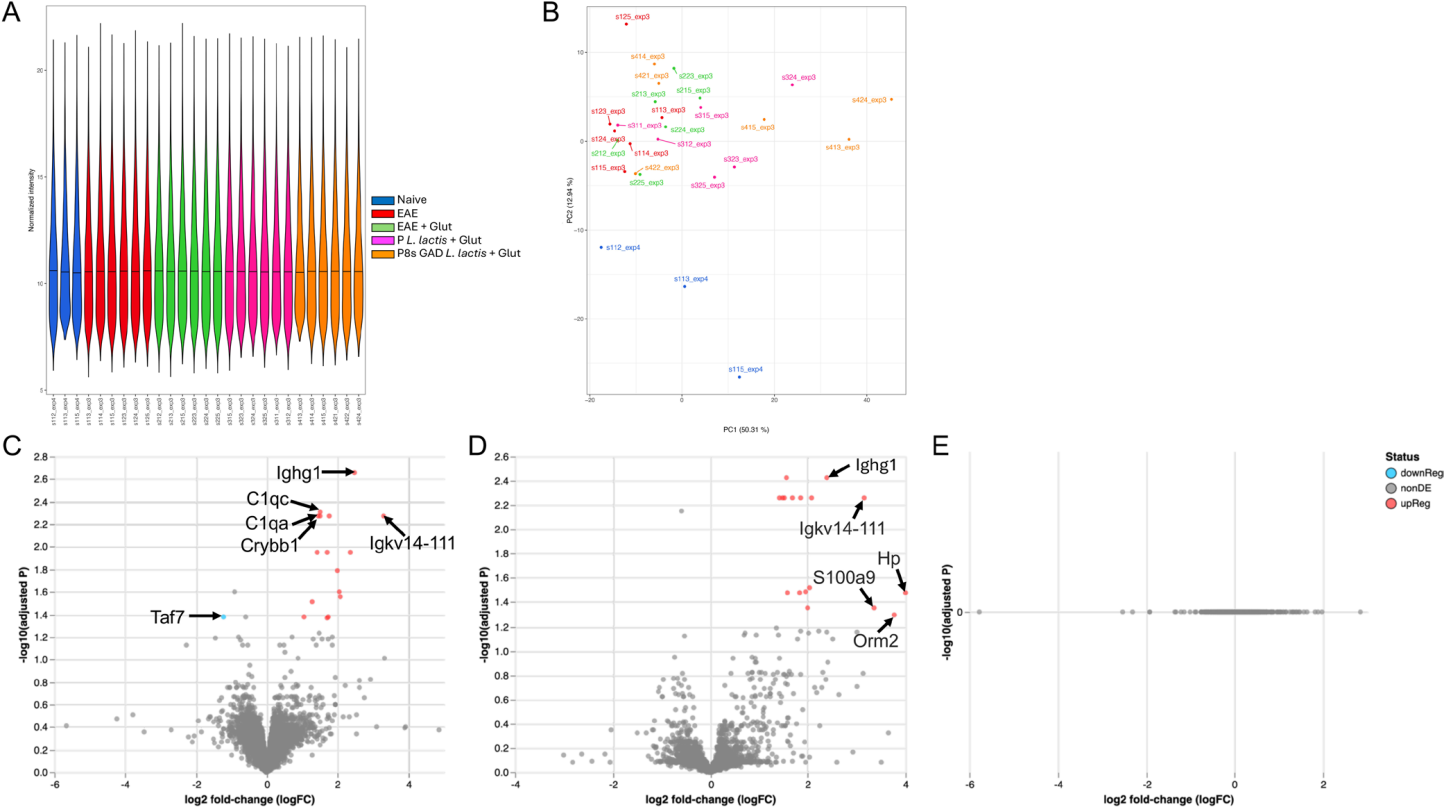
Proteomics analysis of whole brains of EAE mice. (A) Normalized intensity of all samples tested. (B) Principal component analysis and distribution of all samples. (C–E) Volcano plots of the brain proteome of (C) EAE compared to naïve Jax mice; (D) EAE + GA compared to naïve Jax mice; (E) EAE compared to EAE + GA Jax mice. In each plot, 5 up‐ and down‐regulated proteins with the lowest and highest log2 fold‐change (Log_2_FC) and significant FDR value.

To assess whether the oral treatment with P8s‐GAD *L. lactis* and P‐ *L. lactis* would impact the CNS, we performed a proteomics analysis of the whole brains of EAE mice. We compared brains from untreated EAE mice, EAE mice treated with GA, and EAE mice treated with GA and P‐ *L. lactis* (P‐ *L. lactis* + GA) or P8s‐GAD *L. lactis* (P8s‐GAD *L. lactis* + GA), since our in vitro experiments show that glutamic acid is required for GABA production by our probiotic candidate. Brains from naïve mice were also compared. In total, the proteomics analysis identified 9049 proteins across all samples. Figure 7A shows all samples compared and their normalized intensity, indicating homogeneity across samples. Figure 7B illustrates the principal component analysis and distribution of all samples, indicating a distinct protein pattern in naïve versus all EAE groups. Table 5 depicts the number of proteins up, down, and unchanged for each comparison after differential analysis (raw *p*‐value and FDR‐adjusted *p*‐value).

**TABLE 5:** Number of proteins up, down, and unchanged after differential analysis.

Treatment^a^	Significant raw *p*‐value			Significant FDR		
	Down	Unchanged	Up	Down	Unchanged	Up
EAE vs. Naïve	36	8856	105	1	8980	16
EAE + GA vs. Naïve	17	8878	104	0	8892	17
EAE + GA vs. EAE	4	9034	6	0	9044	0
P‐ *L. lactis* vs. Naïve	12	8872	118	0	8982	20
P‐ *L. lactis* vs. EAE	18	9005	23	0	9046	0
P‐ *L. lactis* vs. EAE + GA	3	9039	7	0	9049	0
P8s‐GAD *L. lactis* vs. Naïve	6	8810	183	0	8884	115
P8s‐GAD *L. lactis* vs. EAE	102	8856	85	0	9042	1
P8s‐GAD *L. lactis* vs. EAE + GA	32	8988	27	0	9047	0
P8s‐GAD *L. lactis* vs. P‐ *L. lactis*	1	9036	12	0	9049	0

^a^ GA: Glutamic acid (200 mM). P‐ *L. lactis* and P8s‐GAD *L. lactis* is used to describe EAE mice treated with either strain and that were supplemented with 200 mM GA.

We first evaluated the impact of EAE in brain proteomics by comparing EAE with naïve mice (Figure 7C). EAE induction resulted in the reduction of one (Taf7) and the increase of 16 proteins compared to naïve mice (Table 5 and Figure 7C). When considering *p* values, 36 proteins were down in EAE mice, while 105 were overexpressed (Table 5). The administration of glutamic acid to EAE mice (EAE + GA) resulted in a similar number of proteins with differential abundance (none up and 17 down, compared to naïve; 17 down and 104 up when using raw *p* values; Figure 7D, and Table 5). The administration of GA to EAE mice did not result in any changes in the brain proteomics profile by significant FDR, and only 4 proteins were overexpressed, and 6 were downregulated in the brains of EAE + GA mice compared to EAE mice (Figure 7E).

**FIGURE 8:**
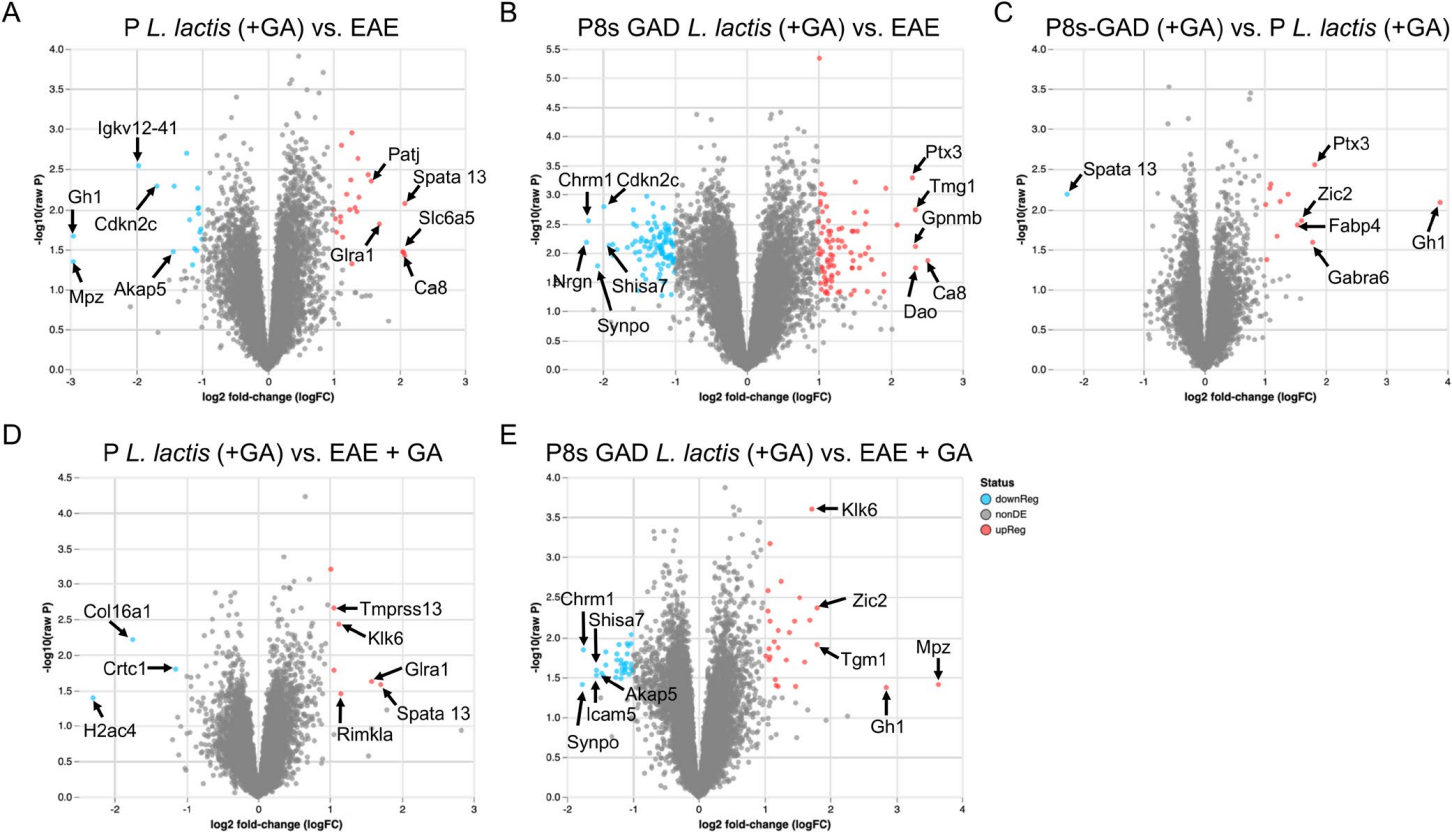
Proteomics analysis of whole brains of EAE mice with P‐ *L. lactis* and P8s‐GAD *L. lactis* or medium supplemented with GA. Proteomics profile of brains from untreated EAE mice, EAE mice treated with GA, and EAE mice treated with GA and P‐ *L. lactis* (P‐ *L. lactis* + GA) or P8s‐GAD *L. lactis* (P8s‐GAD *L. lactis* + GA). (A) Volcano plots of the brain proteome of (A) P‐ *L. lactis* + GA‐treated EAE mice compared to EAE mice; (B) P8s‐GAD‐ *L. lactis* + GA‐treated EAE mice compared to EAE mice; (C) P8s‐GAD‐ *L. lactis* + GA‐treated EAE mice compared to P‐ *L. lactis* + GA mice. (D) P‐ *L. lactis* + GA‐treated EAE mice compared to EAE + GA mice; and (E) P8s‐GAD‐ *L. lactis* + GA‐treated EAE mice compared to EAE + GA mice. In each plot, 5 up‐ and down‐regulated proteins with the lowest and highest log_2_ fold‐change (Log_2_FC) and significant raw *p*‐values.

Next, we compared the effects of administering P‐ *L. lactis* and P8s‐GAD *L. lactis* (both supplied with 200 mM GA) on the brain proteomics profile of mice with those of naïve, EAE, and EAE + GA mice. Comparisons between the bacterial strains and naïve are shown in Figure S17, and protein numbers are depicted in Table 5. Compared to EAE, the proteomic profile of P‐ *L. lactis* ‐treated mice showed 18 proteins downregulated and 23 upregulated, respectively, by raw *p*‐value significance (Table 5, and Figure 8A, with the top 5 down‐ and up‐regulated proteins shown). The top 5 downregulated proteins in P‐ *L. lactis* ‐treated were Myelin protein P0 (Mpz), Somatotropin (Gh1), Immunoglobulin kappa chain variable 12–41 (Igkv12‐41), Cyclin‐dependent kinase 4 inhibitor C (Cdkn2c), and A‐kinase anchor protein 5 (Aka5), and the top 5 upregulated were Carbonic anhydrase‐related protein (Ca8), Spermatogenesis‐associated protein 13 (Spata13), Sodium‐ and chloride‐dependent glycine transporter 2 (Slc6a5), Glycine receptor subunit alpha‐1 (Gira1), and InaD‐like protein (Patj). The profile of P8s‐GAD *L*. *lactis*‐treated mice showed a more drastic change, compared to EAE, with 102 proteins downregulated and 85 upregulated (Table 5, and Figure 8B, with the top 5 down‐ and up‐regulated proteins shown), and one protein (von Willebrand factor (Vwf)) was significantly upregulated by FDR values.

Compared to EAE + GA, P‐ *L. lactis* ‐treated mice showed no changes in protein differential abundance by FDR significance, while 3 were downregulated (Histone H2A type 1‐B (H2ac4), CREB‐regulated transcription coactivator 1 (Crtc1), and Collagen alpha‐1 chain (Col16a1)) and 7 proteins upregulated (Top 5: Spermatogenesis‐associated protein 13 (Spata13), Glycine receptor subunit alpha‐1 (Gira1), N‐acetylaspartylglutamate synthase A (Rimkla), Kallikrein related‐peptidase 6 (Klk6), and Transmembrane protease serine 13 (Tmprss13)), by raw *p*‐value significance (Figure 8D and Table 5). Similarly, treatment with P8s‐GAD *L. lactis* resulted in no changes in protein profiles by FDR significance compared to EAE mice; however, the number of proteins down‐ and up‐regulated in treated mice was substantially higher: 32 down and 27 up (Figure 8E and Table 5). The top 5 proteins downregulated in P8s‐GAD *L. lactis* were Synaptopodin (Synpo), Muscarinic acetylcholine receptor M1 (Chrm1), Intercellular adhesion molecule 5 (Icam5), Protein shisa‐7 (Shisa7), and A‐kinase anchor protein 5 (Akap5). The top 5 proteins upregulated in P8s‐GAD *L. lactis* were Somatotropin (Gh1), Mielin protein P0 (Mpz), Zinc finger protein ZIC 2 (Zic2), Kallikrein related‐peptidase 6 (Klk6), and Protein‐glutamine gamma‐glutamyltransferase K (Tgm1).

Finally, we compared the proteomics profile of the brains of mice treated with P8s‐GAD *L. lactis* and P‐ *L. lactis* . Again, no changes in protein profiles were identified (Figure 8C), while one protein was down and 12 were up in P8s‐GAD *L. lactis* ‐treated mice compared to P*‐L. lactis*‐treated mice. Table 6 depicts the proteins down‐ and up‐regulated in the brains of P8s‐GAD *L. lactis* ‐treated mice compared to P‐ *L. lactis* ‐treated mice. While only one protein was downregulated in P8s‐GAD *L. lactis* ‐ compared to P‐ *L. lactis* ‐treated mice (Spata13), the list of proteins identified as upregulated in P8s‐GAD *L. lactis* ‐treated mice includes hormones (Somatotropin (Gh1)), immune system components (Pentraxin‐related protein PTX3 (Ptx3), Histocompatibility 2, Q region locus 1 (H2‐Q1), Cytochrome b‐245 light chain (Cyba, Ebf1)), neuronal proteins (gamma‐aminobutyric acid receptor subunit alpha‐6 (Gabra6), Zinc finger protein ZIC 2 (Zic2), Complexin 4 (Cplx4)), metabolic proteins (fatty acid‐binding protein 4 (Fabp4)), and a protein of unknown function (Fam124a) (Table 6). Thus, compared to the treatment with P‐ *L. lactis* , the GABA‐producing bacterium could enhance a protein pattern associated with varied functions, indicating no common biological pathway, structural motif, or disease association that unites them. Inflammatory cytokines associated with EAE were detected in the proteomic profiles; however, no significant differences between groups were observed.

**TABLE 6:** Proteins down and up‐regulated in P8s‐GAD *L. lactis* compared to P‐ *L. lactis* ‐treated EAE mice (+GA^a^).

	Protein group	Log CF	Raw *p*	FDR adj *p*
Down (P8s vs. P *L. lactis* )				
Spata13	Q5DU57	−2.2567	0.0065	> 0.05
Up (P8s vs. P *L. lactis* )				
Gh1	P06880	3.8851	0.0082	> 0.05
Ptx3	P48759	1.8125	0.0027	> 0.05
Gabra6	P16305	1.7747	0.0259	> 0.05
Igkv4‐70	A0A0B4J1I5	1.6007	0.0139	> 0.05
Zic2	Q62520	1.5392	0.0158	> 0.05
Fabp4	P04117	1.3863	0.0064	> 0.05
H2‐Q1	O19441	1.2405	0.0079	> 0.05
Cyba	Q61462	1.1884	0.0215	> 0.05
Ebf1	Q07802	1.0873	0.0048	> 0.05
Fam124a	D3Z5V4	1.0707	0.0054	> 0.05
Cplx4	Q80WM3	1.0231	0.0433	> 0.05
Ig H chain V region 914	P18527	1.0071	0.0087	> 0.05

^a^ GA: Glutamic acid (200 mM). P‐ *L. lactis* and P8s‐GAD *L. lactis* is used to describe EAE mice treated with either strain, and that were supplemented with 200 mM GA.

## 4 Discussion

This work tested the protective effects of P8s‐GAD *L. lactis* , a GABA‐producing bacterial strain, against experimental CNS demyelinating inflammation. We also tested whether the oral treatment with the bacterium would impact the microbiota and mycobiota composition, and whether the treatment affected the brain proteome of EAE mice.

Both intestinal bacteria and host enzymes play a role in the metabolism of GABA and L‐glutamate. GABA is an inhibitory neurotransmitter and key regulator of the gastrointestinal (GI) tract function, while L‐glutamate (Glu) is an excitatory neurotransmitter. Decarboxylation of Glu by the enzyme glutamic acid decarboxylase (GAD) produces GABA. We recently published the construction and characterization of an engineered *Lactococcus lactis* strain with increased secretion of the neurotransmitter GABA due to the insertion of an extra plasmid‐based copy of the *L. lactis* IL1408 *gadCB* operon, where *gadB* encodes glutamic acid decarboxylase, and *gadC* encodes the glutamic acid‐GABA antiporter [20]. Our work demonstrated that in the presence of glutamic acid (GA), substrate of the GAD enzyme, P8s‐GAD *L. lactis* produced significantly more GABA in vitro than the control strain. Probiotics can be administered to alleviate disease due to the role of the gut microbiome in regulating the immune system and modulating human diseases. It has been shown that in MS patients, GABA levels are decreased and inversely correlate with neurological manifestations and inflammation. Immune cells' exposure to GABA shifts their function toward anti‐inflammatory responses [15, 16, 33], and GABAergic compounds are protective in the EAE model [15, 34]. Furthermore, we have shown that neuroinflammation reduces the relative abundance of GABA‐producing bacteria during disease [1, 2, 3]. Others documented decreased GABA levels in the stool of MS patients [35]. We hypothesized that oral treatment by gavage with P8s‐GAD *L. lactis* would protect against EAE and modulate gut dysbiosis associated with the disease. Our findings suggest that P8s‐GAD *L. lactis* did not confer protection in mice with different microbial makeup. While the bacterial strain was protective against EAE in Env mice, it failed to protect consistently in Jax mice (Figure 1A–D). Our initial studies also suggest that adding the *gadCB* operon to *L. lactis* IL1408 is not sufficient to trigger protective effects against severe neuroinflammation in EAE mice, even when GA (200 mM), the GAD substrate, is co‐administered (Figure 1E–H). While the GM17 medium used to grow our bacteria contains 0.66 mM of L‐glutamic acid [32], the constant exposure to 200 mM in drinking water did not impact EAE's clinical severity (Figure S8).

We published a microbiome analysis of Env and Jax mice that reported significant differences as the disease progressed [2]. We also observed remarkable differences in Env vs. Jax in the predicted functional metabolic and cellular process pathway abundances [25]. Although differences in the microbiome makeup of Env and Jax mice could be proposed as a critical factor modulating protection by a given probiotic, genetic drift occurring over generations could also explain the differences observed in our study [36]. It is possible that different concentrations of glutamic acid in diets used could affect the protection studies: Teklad 22/5 rodent diet (Envigo) was used for the experiment shown in Figure 1A–D, with 3.6% L‐glutamic acid content, while Lab Rodent Diet 5001 (LabDiet Inc.) was used in experiments shown in Figure 1E–H, with a 4.54% L‐glutamic acid content. However, as indicated above, supplementation with GA did not improve the protective effects of P8s‐GAD *L. lactis* (Figure 1E–H).

We have previously shown that in the context of EAE, severity and the timeline determine the changes in the microbiota composition [3]. Although the most significant shift in beta diversity was observed between D0 and D14 (Figure 2D), EAE treatment with GABA‐producing P8s‐GAD *L. lactis* reduced alpha diversity by the end of the experiment (D28) compared to Medium‐treated controls (Figure 2B). Members of the phylum Bacteroidota were significantly increased in P8s‐GAD *L. lactis* ‐treated mice compared to Medium mice; by contrast, Firmicutes were significantly reduced at the end of the experiment (D28) (Figure S2B). The Firmicutes: Bacteroidetes (F:B) ratio has been proposed as a bioindicator for dysbiosis in the context of EAE [37] and as a parameter to assess the efficacy of a probiotic approach [38]. However, despite a trend suggesting a reduction in the F:B ratio in mice treated with P8s‐GAD *L. lactis* , no statistical differences were found compared to the Medium group of mice. No changes were observed in mice treated with P‐ *L. lactis* compared to controls or P8s‐GAD *L. lactis* (Figure S2). The co‐administration of GA with the bacterial strains did not have an impact on these results (Figure S13).

At D28, P8s‐GAD *L. lactis* ‐treated mice showed reduced relative abundances of the genera *Lachnospiraceae* UCG‐004, *Lachnospiraceae* UCG‐006, *Bacteroides*, and *Oscillibacter*. In contrast, *Rombutsia* and *Parvibacter*'s abundances were increased compared to Medium controls and P‐ *L. lactis* ‐treated mice. *Lachnospiraceae* UCG‐004, *Lachnospiraceae* UCG‐006, and *Oscillibacter* were also reduced in samples from P‐ *L. lactis* ‐treated mice compared to Medium‐treated controls. The role of *Lachnospiraceae* in inflammation remains unclear, with some reports highlighting anti‐inflammatory effects [39]. *Oscillibacter* has been positively correlated with increased gut permeability, which could, in turn, trigger inflammation [40]. The increase observed in P8s‐GAD *L. lactis* ‐treated mice in *Rombutsia*, a Gram‐positive bacterium previously proposed as a constituent in probiotics [41], was shown in D28. The genus *Parvibacter*, containing one species (*Parvibacter caecicola*), was shown to be increased in P8s‐GAD *L. lactis* compared to P‐ *L. lactis* (not significant difference compared to medium control; Figure S5). The bacterium has been associated with chronic inflammation associated with AhR‐depleted diet administration [42]. By contrast, members of the genus *Bacteroides* were reduced in P8s‐GAD *L. lactis* compared to P*‐L. lactis*‐treated mice. Although members of Bacteroides, such as polysaccharide A‐producing *B. fragilis* , are well‐known microbiota regulators of intestinal inflammation [34, 35, 43, 44] and neuroinflammation [45, 46, 47, 48], the genus also contains members associated with exacerbated inflammatory responses [49]. Further studies of gut metabolomics, gut microbiome metatranscriptomics, and in vivo functional studies will elucidate the biological importance of the changes that the GABA‐producing P8s‐GAD *L. lactis* promote in the bacterial compartment of the gut of EAE mice.

The gut mycobiome of people with MS differs from that of healthy individuals, with increased alpha diversity [50] and altered differential abundances with enhanced *Saccharomyces* and *Aspergillus* species [51]. The impact of experimental disease in the fungal compartment of the animals' gut has yet to be explored in detail. The oral administration of *Candida kefyr* is protective against EAE in mice by reducing Th17 cell responses and increasing tolerogenic CD103^+^ dendritic cells and regulatory Tregs [52]. In addition, the treatment resulted in increased Lactobacillales and decreased Bacteroides compared to controls, suggesting that the effects could also be mediated by alterations in the gut microbiota [52]. However, our study might be the first to assess the direct impact of EAE induction and progression on the mycobiome of mice. The treatment with the bacterial strains promoted significant changes in the mycobiome composition (D‐7 vs. D0). Furthermore, EAE modified the mycobiome throughout the experiment (D0 vs. D14 and D14 vs. D28; Figure 5D). P‐ *L. lactis* and P8s‐GAD *L. lactis* treatments only further modified the mycobiome composition during the early stages of the disease between D0 and D14. Possibly due to the small number of samples used, our analysis did not identify any taxa differentially present between experimental groups and timepoints.

When GA was co‐administered with P8s‐GAD *L. lactis* , the microbiome changes were mostly observed between days 14 and 21 (Figure 5C). It is important to note that the alpha diversity quantification showed an increase in richness and evenness at the beginning of the study (Figure 5B), possibly because of the small number of samples analyzed and interindividual variability. Interestingly, the number of bacterial families and genera significantly altered in response to the GA + P8s‐GAD *L. lactis* co‐treatment was reduced compared to what was observed in the absence of GA (Tables 3 and 4, Figure S14). The impact of co‐administration of GA with P8s‐GAD *L. lactis* resulted in a significant increase in *Akkermansia* and a reduction in an unknown genus of Clostridia‐UCG014, at days 0 and 14 (Figure S14). At the mycobiome level, P8s‐GAD *L. lactis* + GA co‐administration resulted in the overabundance of an unknown fungal taxa (Figure S15). These findings suggest that the GA addition to the treatment with P8s‐GAD *L. lactis* did not substantially modify the impact of P8s‐GAD *L. lactis* on microbiome and mycobiome compositions.

Given the lack of consistent protection against EAE induced by P8s‐GAD *L. lactis* in Jax mice, we questioned whether the bacterium could have detectable effects on the brains of diseased mice. We performed proteomics analyses in brains isolated from mice used for the experiment shown in Figure 1E–H. Despite the limited sample size, the number of proteins up‐ and down‐regulated by raw *p* values shown in Table 5 indicates that P8s‐GAD *L. lactis* treatment had a greater impact on the brains of EAE mice than Medium and P‐ *L. lactis* treatments. Interestingly, Gabra6, the alpha‐6 subunit of the GABA type A (GABA_R_A) receptor, was upregulated in P8s‐GAD *L. lactis‐*treated mice, suggesting that the treatment with the GABA‐producing *L. lactis* could indeed positively regulate GABAergic pathways in the brains of EAE mice. More adequately powered studies will be needed to fully elucidate the impact of P8s‐GAD *L. lactis* on the CNS of diseased mice. However, our data already suggest that administering GABA‐producing *L. lactis* could alter the proteome profile of these mice.

Our study has important limitations to consider: first, we cannot replicate the experiment performed in Env mice since the group was moved to a different laboratory at a different university, with different animal facilities, environmental conditions, and regulations; second, stool samples from Env mice are not available which makes impossible the analysis of the gut microbiota of mice treated with the bacterial strains; and third, all experiments were done in female mice. Despite these limitations, our work suggests the need to control the source of mice and housing conditions when considering probiotics as a novel avenue to treat neuroinflammation. Furthermore, our GABA‐producing P8s‐GAD *L. lactis* strain may be considered for other clinical applications, including pathologies associated with imbalances in neurotransmitter levels.

## Author Contributions

K.H. was responsible for executing the in vivo experiments. A.L., S.N., D.B., K.F., and H.K. contributed to sample collections. A.C., J‐.B.R., and K.M.G. contributed to the conceptualization of the project, the revision of the article, and the literature search. T.O.K. supervised microbiome and mycobiome analysis and prepared figures. J.O‐.R. was responsible for research design, article preparation/revision, and supervisory support.

## Funding

This work was supported by the National Institutes of Health (NIH) (grant R15NS107743), the Institutional Development Awards (IDeA) from the National Institute of General Medical Sciences of the National Institutes of Health under Grants #P20GM103408, #P20GM109095, and #P20GM148321.

## Conflicts of Interest

T.O.K. is employed by AG1, a company specializing in pre‐ and probiotics and nutritional supplements. The rest of the authors (K.H., A.L., S.N., D.B., K.F., H.K., A.C., J.‐B.R., K.M.G., and J.O.‐R.) declare no conflicts of interest regarding the publication of this article.

## Supporting information


**Data S1:** fba270085‐sup‐0001‐supinfo.pdf.

## Data Availability

The data that support the findings of this study are openly available in Dryad. Microbiome sequencing raw data and metadata files are stored at https://doi.org/10.5061/dryad.66t1g1kbv; Mycobiome sequencing raw data and metadata files are stored at https://doi.org/10.5061/dryad.7d7wm3866. Proteomics raw data and metadata files are stored at https://doi.org/10.5061/dryad.x0k6djj04.
