# Supplementary material for: Impact of a GABA‐Producing Lactococcus lactis on Microbiota and Mycobiota During CNS Inflammatory Demyelination
Source: FASEB Bioadv. 2026 Jan 7;8(1):e70085. doi: 10.1096/fba.2025-00082 (PMC12777695; doi:10.1096/fba.2025-00082)
Supplement: Supplementary file 1 — Data S1: fba270085‐sup‐0001‐supinfo.pdf. [file FBA2-8-e70085-s001.pdf]

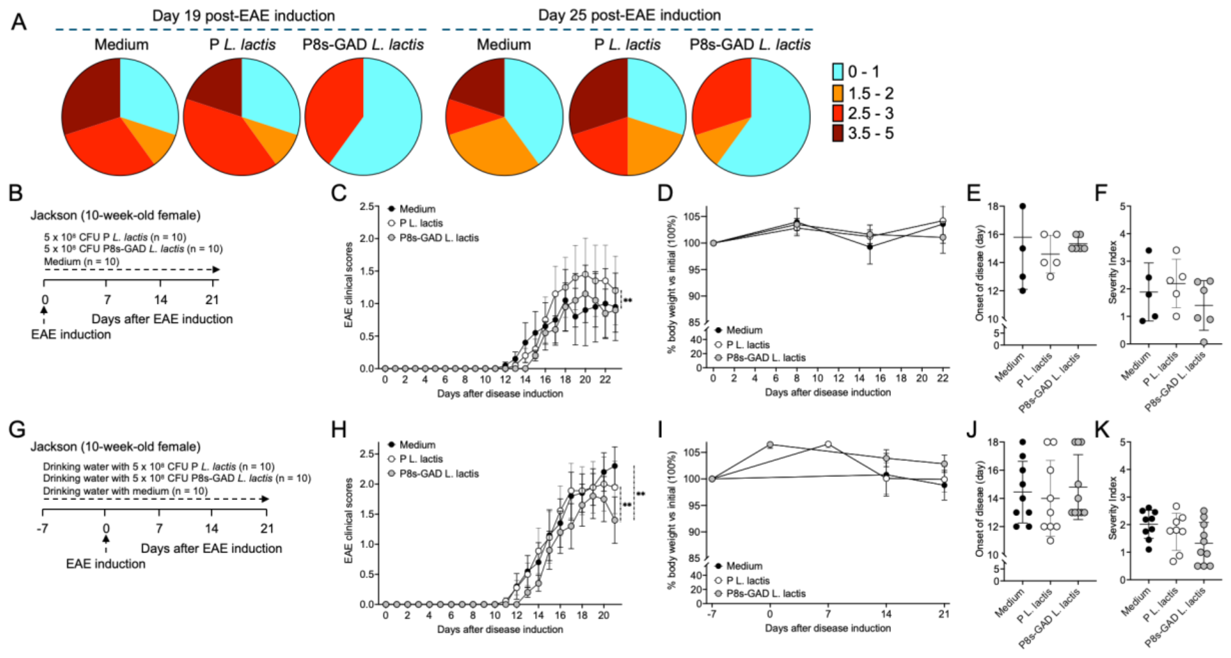

Supplemental Figure 1. A) Distribution of EAE clinical scores on D19 and end of the experiment (D25). B-F) Jax mice were treated by oral gavage with frozen 100 mL of 5x10^{8} CFU of P8s-GAD *L. lactis* or P-*L. lactis*, or 100 microL of GM17 medium (Medium), five consecutive days per week from the day of EAE induction (D0) until the end of the experiment; G-K) Mice were treated with 5x10^{9} CFU/ml of P8s-GAD *L. lactis* or P-*L. lactis*, or 100 mL of GM17 medium (Medium) *ad libitum*, starting seven days before EAE induction (D-7) until the end of the experiment; B and G: Graphical representation of each experiment. C and H: Daily clinical scores represented as mean +/- SEM, analyzed by repeated measures of ANOVA & Tukey's test. D and I: Body weight percentage compared to the initial value at D0, represented as mean +/- SEM, analyzed by repeated measures of ANOVA & Tukey's test. E and J: EAE clinical onset depicted as the mean +/- SEM and compared by Mann-Whitney test. F and K: EAE clinical severity, calculated as the cumulative scores divided by the number of days with EAE scores per mouse, depicted as the mean +/- SEM, and compared by Mann-Whitney test. *, P < 0.05; **, P < 0.01. ***, P < 0.001. n = 10 per group.

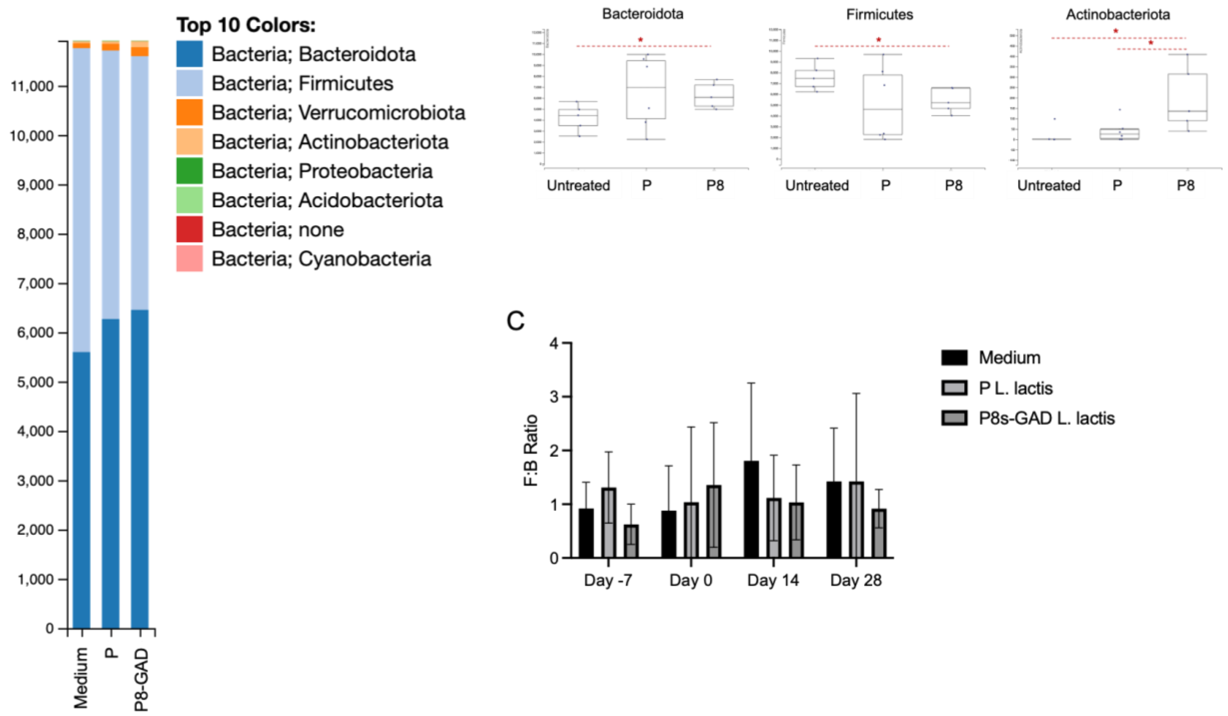

Supplemental Figure 2. A) Barplots representing the relative abundances of the gut microbiome at the phylum level on D28; B) Differential abundance at the Phylum level between groups on D28 in mice treated with medium (labeled as untreated), P L. lactis, and P8s-GAD L. lactis. Non-parametric Wilcoxon Rank-Sum analysis. *, $P < 0.05$. Phylum level Day 28. No differences at the remaining timepoints. C) Firmicutes:Bacteroidetes (F:B) ratio, compared by non-parametric ANOVA, followed by Tukey's multiple comparisons test. All comparisons resulted in non-significant ($p > 0.05$).

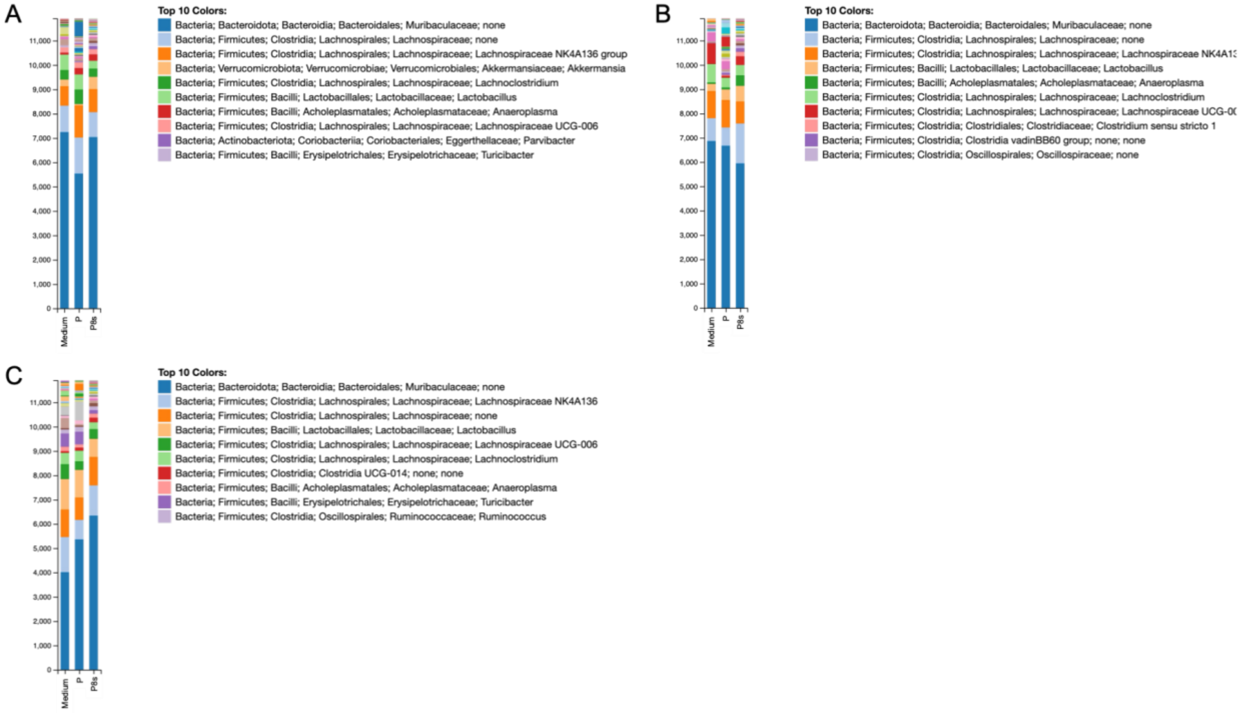

Supplemental Figure 3. Barplots representing the relative abundances of the gut microbiome at the genus level on A) D-7 (day of beginning of treatments and seven days before EAE induction), B) D0 (day of EAE induction), and C) D14 (14 days after EAE induction).

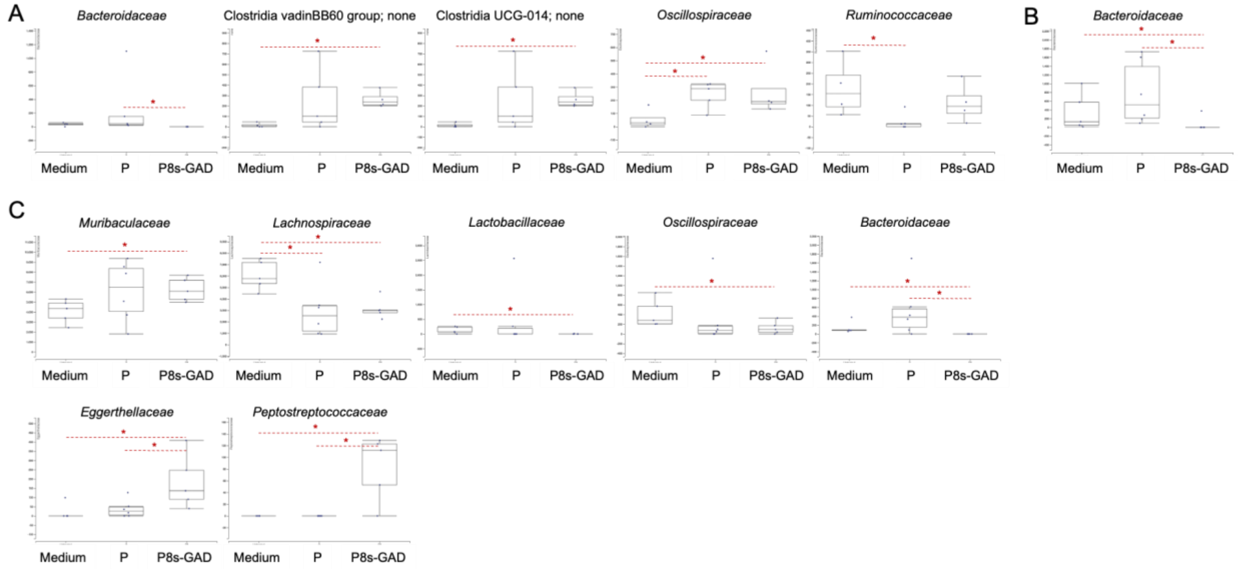

Supplemental Figure 4. P8s-GAD L. lactis treatment impacts the relative abundances of specific families of the gut microbiota. Mice were treated by oral gavage with 100 µL of 5 × 10^8 CFU of P8s-GAD L. lactis or P-L. lactis, or 100 µL of GM17 medium (Medium), five consecutive days per week, starting seven days before EAE induction (D-7) until the end of the experiment. Differential abundance at the family level between groups. A) D0, B) d14, C) D28. Non-parametric Wilcoxon Rank-Sum analysis. *, $p < 0.05$.

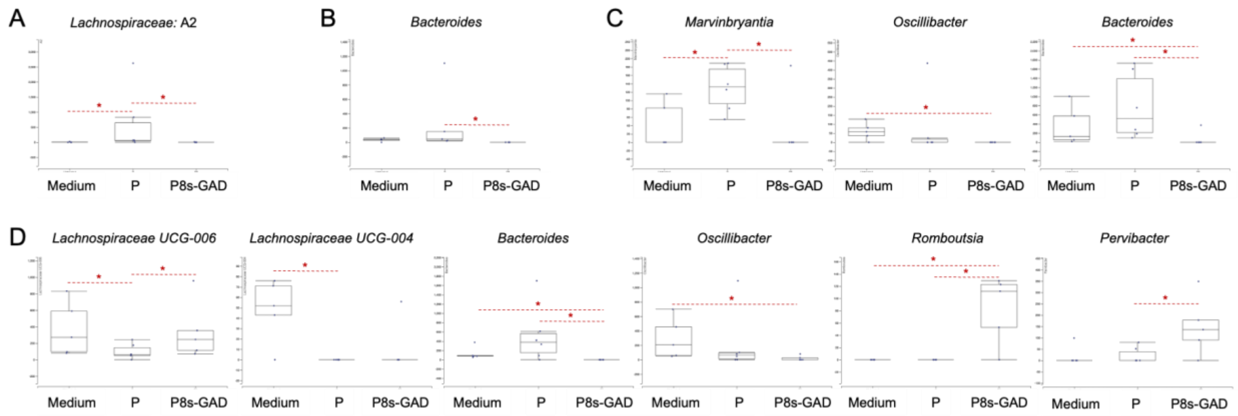

Supplemental Figure 5. P8s-GAD *L. lactis* treatment impacts the relative abundances of specific genera of the gut microbiota. Mice were treated by oral gavage with 100 μL of 5 x 10^8 CFU of P8s-GAD L. lactis or P-L. lactis, or 100 μL of GM17 medium (Medium), five consecutive days per week, starting seven days before EAE induction (D-7) until the end of the experiment. Differential abundance at the family level between groups. A) D-7, B) D0, C) d14, D) D28. Non-parametric Wilcoxon Rank-Sum analysis. *, $p < 0.05$.

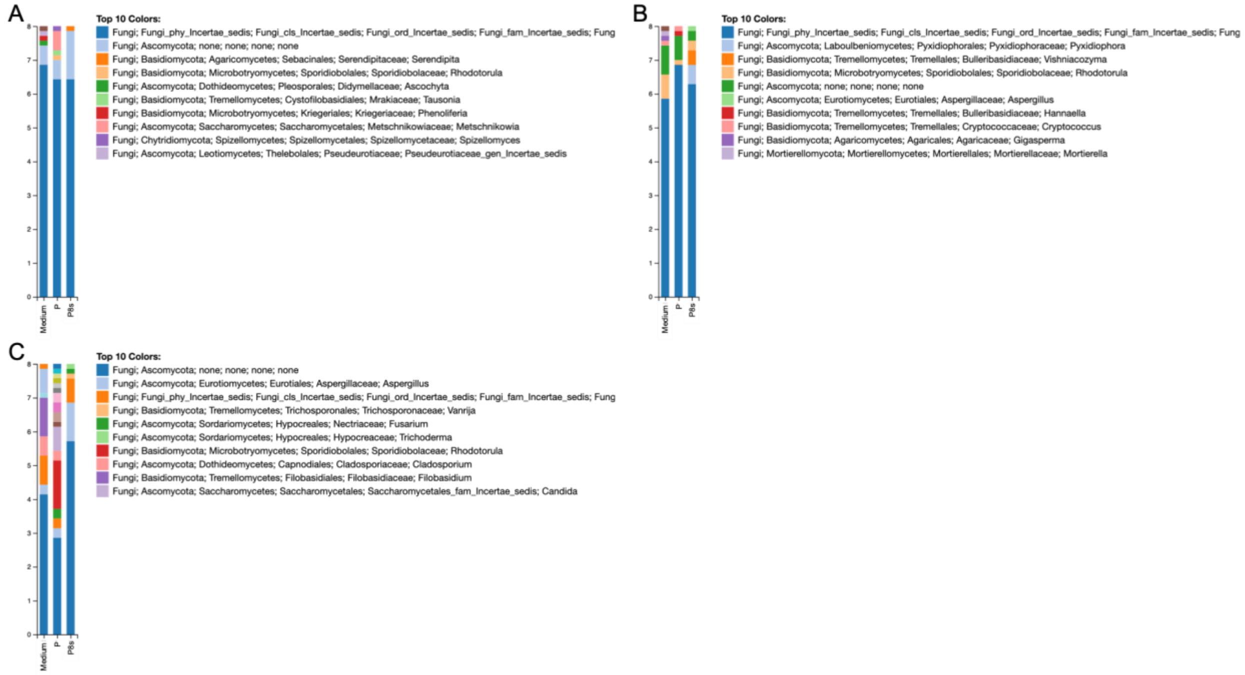

Supplemental Figure 6. Barplots representing the relative abundances of the gut mycobiome at the genus level on A) D-7 (day of beginning of treatments and seven days before EAE induction), B) (day of EAE induction), and C) D14 (14 days after EAE induction).

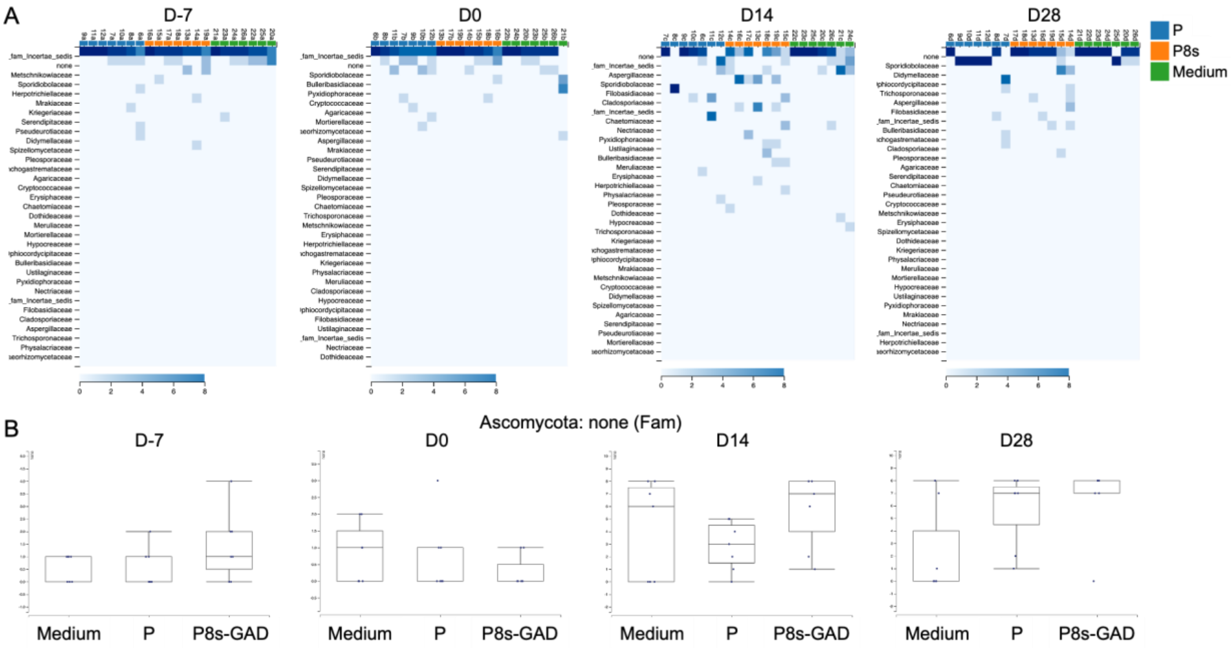

Supplemental Figure 7. Mice were treated by oral gavage with 100 μL of 5 x 10^8 CFU of P8s-GAD L. *lactis* or P-L. *lactis*, or 100 μL of GM17 medium (Medium), five consecutive days per week, starting seven days before EAE induction (D-7) until the end of the experiment. Heatmap plots from D-7, D0, D14, and D28. B) Relative abundance of an unknown family of Phylum Ascomycota at D-7, D0, D14, and D28. Fisher's exact test first, and then the Wilcoxon Rank-Sum.

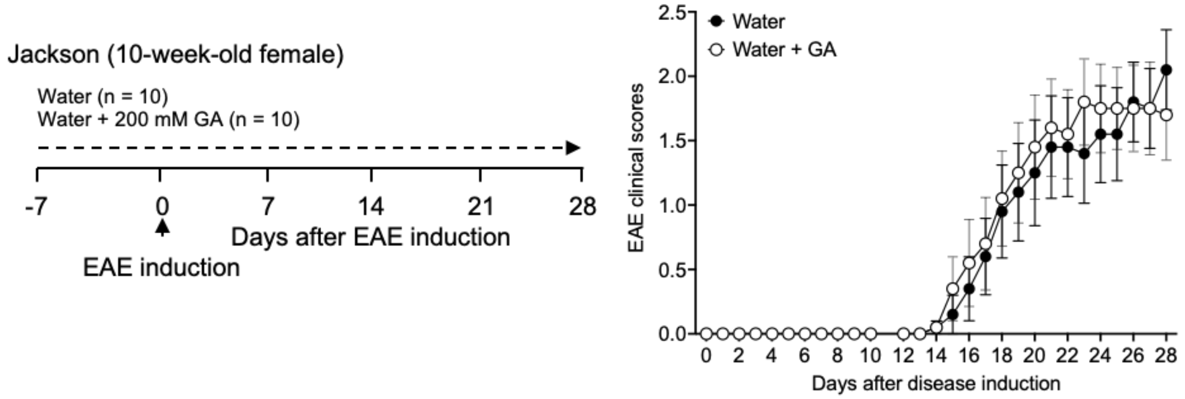

Supplemental Figure 8. Effects of GA on EAE progression. Mice were given 200 mM GA in drinking water from D-7 to D28. F: Graphical representation of experiment. G: Daily clinical scores represented as mean +/- SEM, analyzed by repeated measures of ANOVA & Tukey’s test. n = 10 per group.

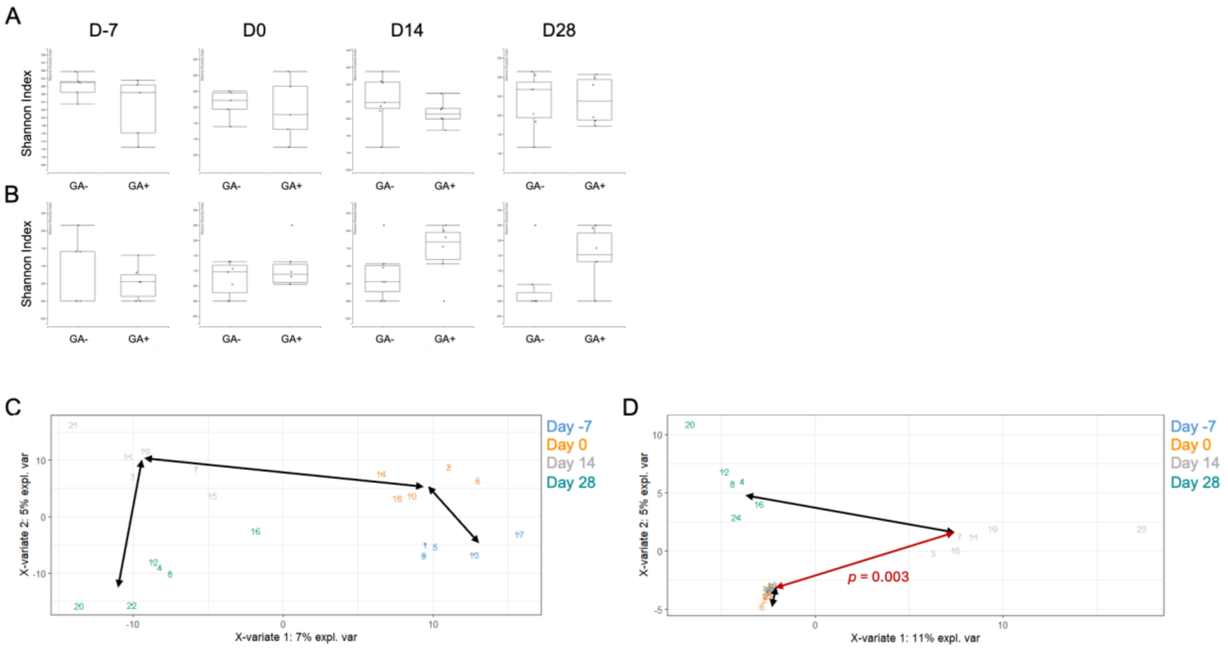

Supplemental Figure 9. Effects of GA supplementation on the composition of microbiome and mycobiome in EAE (comparing EAE mice treated with 200 mM GA administered in Medium or Medium without GA). A) Alpha diversity (Shannon Index) in microbiome (16S rRNA sequencing); B) Alpha diversity (Shannon Index) in mycobiome (ITS sequencing); No significant effects were observed. C) Beta diversity, visualized by two-dimensional Plot of Individuals (PlotInd) with pairs of groups (all timepoints) analyzed with Adonis in microbiome; D) Beta diversity, visualized by two-dimensional Plot of Individuals (PlotInd) with pairs of groups (all timepoints) analyzed with Adonis in mycobiome.

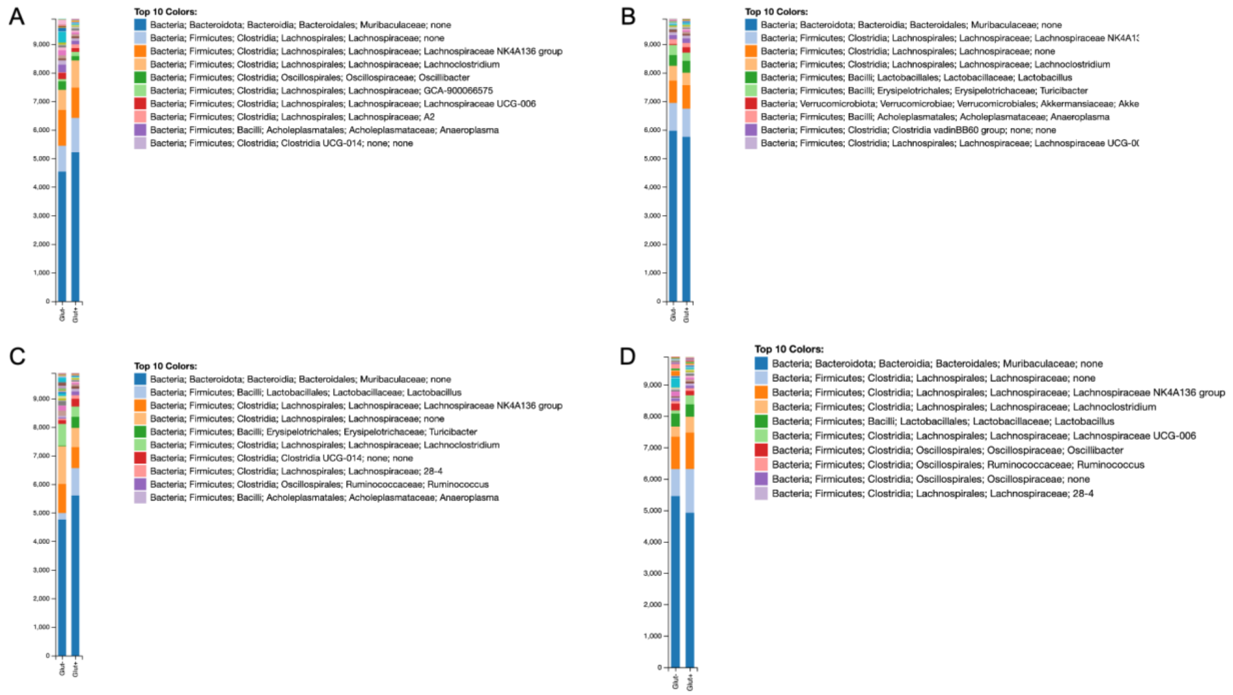

Supplemental Figure 10. Barplots representing the relative abundances of the gut microbiome of mice treated or not with 200 mM glutamic acid (GA), at the genus level on A) D-7 (day of beginning of treatments and seven days before EAE induction), B) D0 (day of EAE induction), C) D14 (14 days after EAE induction), and D) D28 (28 days after EAE induction).

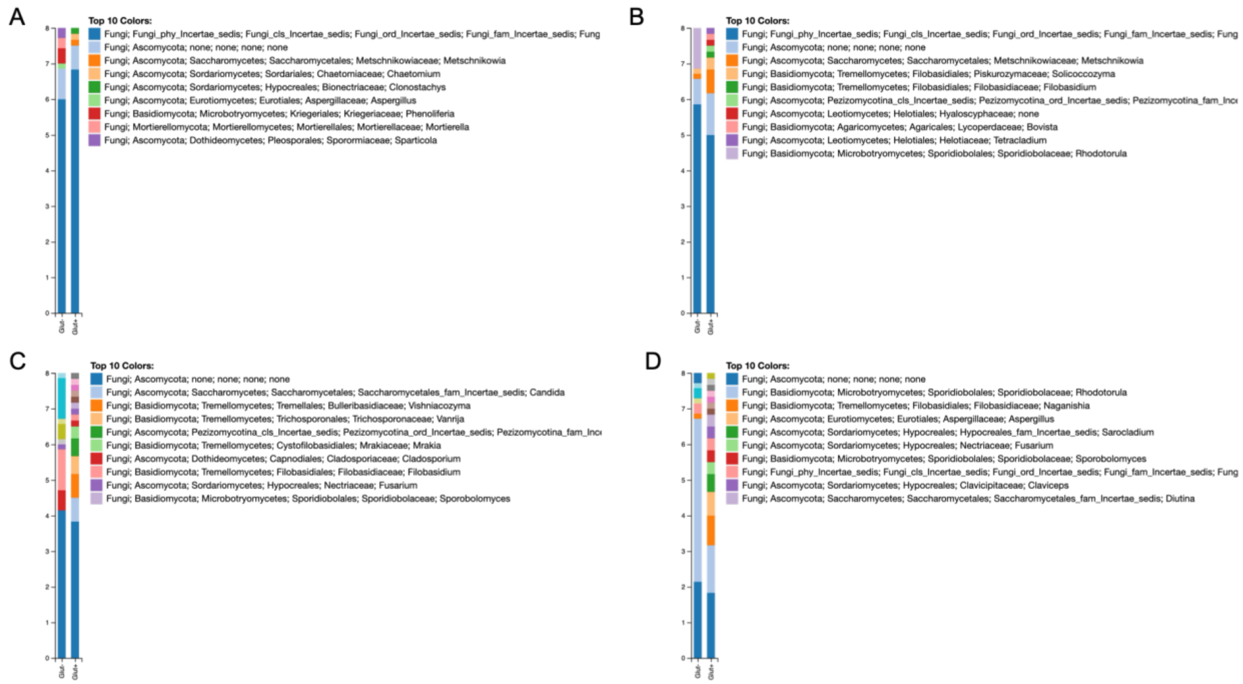

Supplemental Figure 11. Barplots representing the relative abundances of the gut mycobiome of mice treated or not with 200 mM glutamic acid (GA), at the genus level on A) D-7 (day of beginning of treatments and seven days before EAE induction), B) D0 (day of EAE induction), C) D14 (14 days after EAE induction), and D) D28 (28 days after EAE induction).

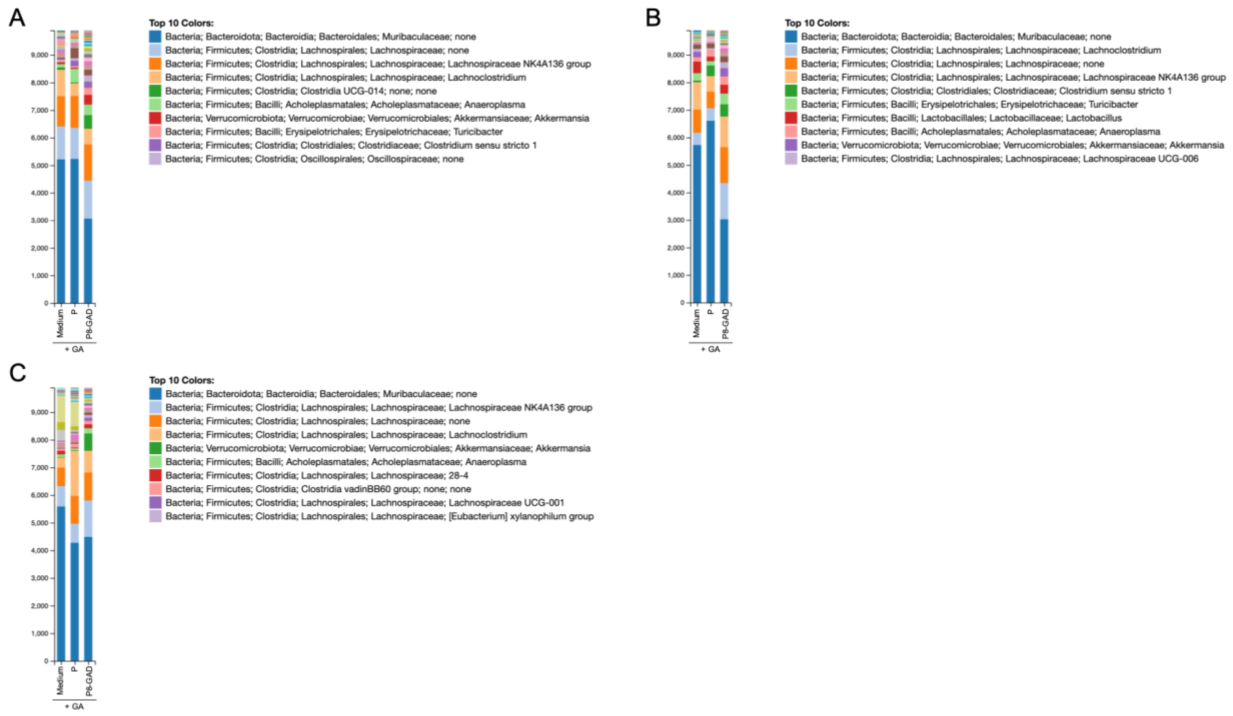

Supplemental Figure 12. Barplots representing the relative abundances of the gut microbiome of mice treated with medium, P L. lactis or P8s-GAD L. lactis, supplemented with 200 mM Glutamic acid (GA) at the genus level on A) D-7 (day of beginning of treatments and seven days before EAE induction), B) D0 (day of EAE induction), and C) D14 (14 days after EAE induction).

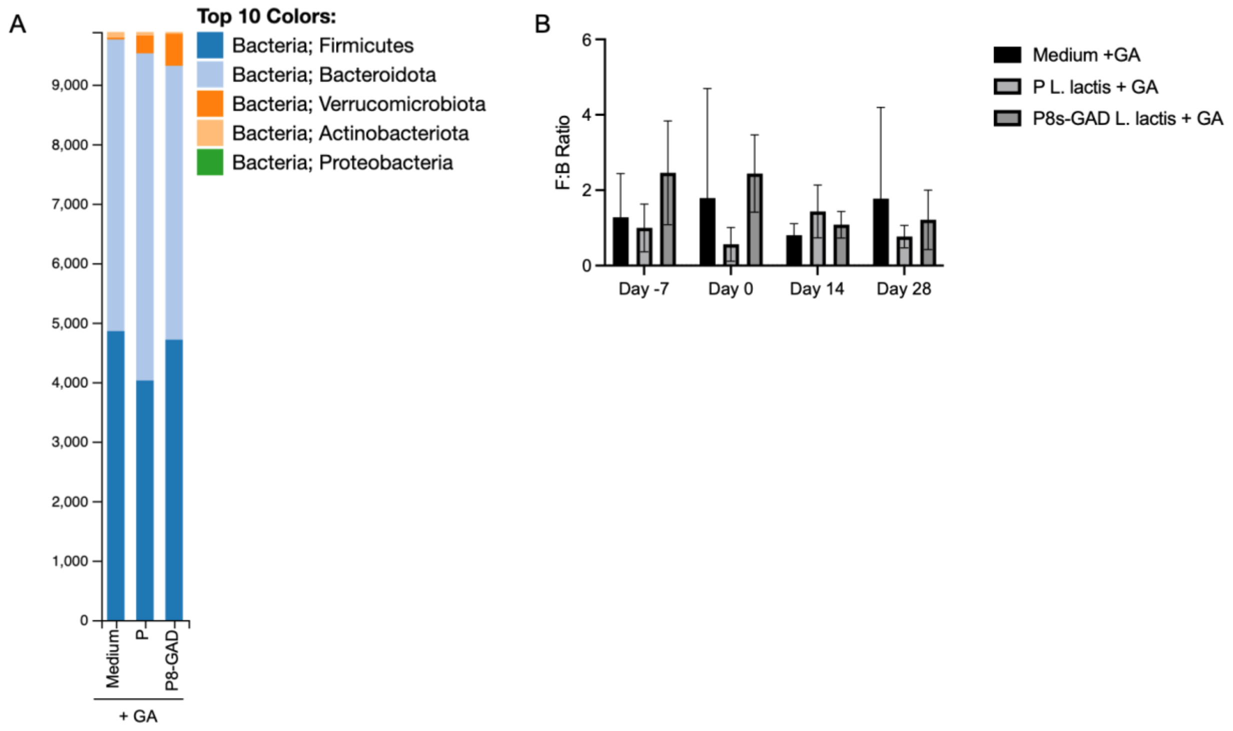

Supplemental Figure 13. A) Barplots representing the relative abundances of the gut microbiome at the phylum level on D28 in mice treated with medium (labeled as untreated), P *L. lactis*, and P8s-GAD *L. lactis*, supplemented with 200 mM glutamic acid (GA). B) Firmicutes:Bacteroidetes (F:B) ratio, compared by non-parametric ANOVA, followed by Tukey's multiple comparisons test. All comparisons resulted in non-significant ($p > 0.05$).

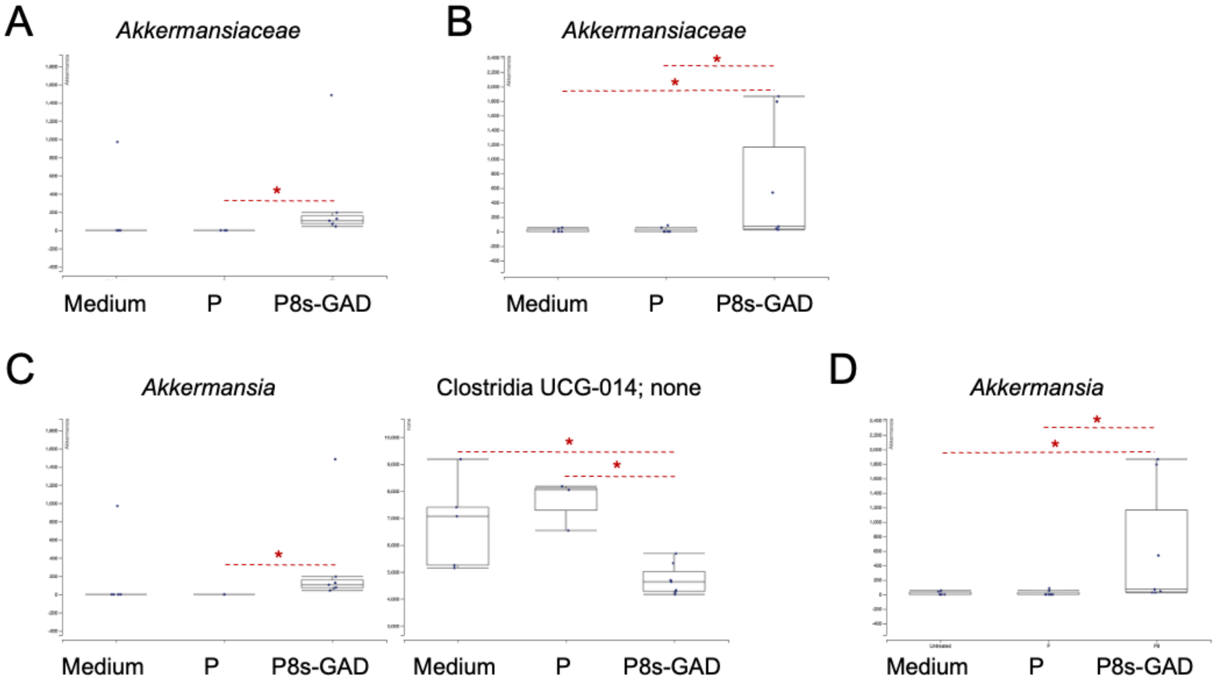

Supplemental Figure 14. Differential abundance at the family and genus level between groups in mice, untreated or treated with P-L. lactis or P8s-GAD L. lactis, supplemented with 200 mM GA. A) Family, D0; B) Family, D14; C) Genus; D0; D) Genus, D14. Non-parametric Wilcoxon Rank-Sum analysis. *, $p < 0.05$. No differences observed at D-7.

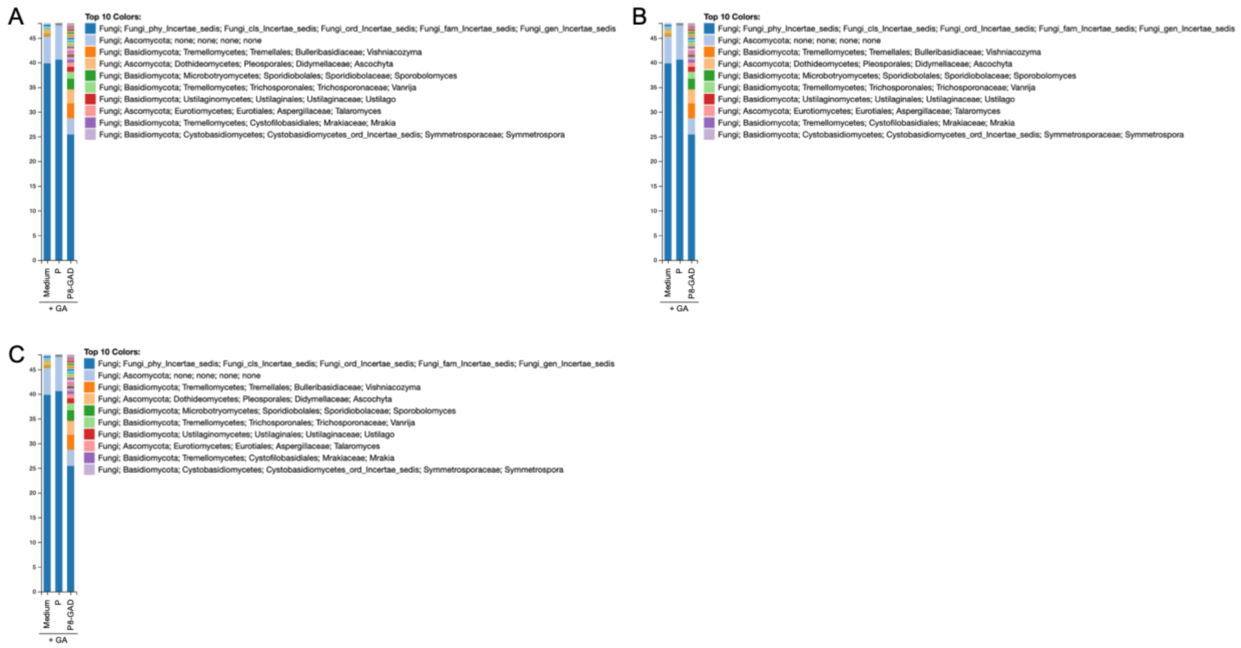

Supplemental Figure 15. Barplots representing the relative abundances of the gut mycobiome of mice treated with medium, P *L. lactis,* or P8s-GAD *L. lactis*, supplemented with 200 mM Glutamic acid (GA) at the genus level on A) D-7 (day of beginning of treatments and seven days before EAE induction), B) D0 (day of EAE induction), and C) D14 (14 days after EAE induction).

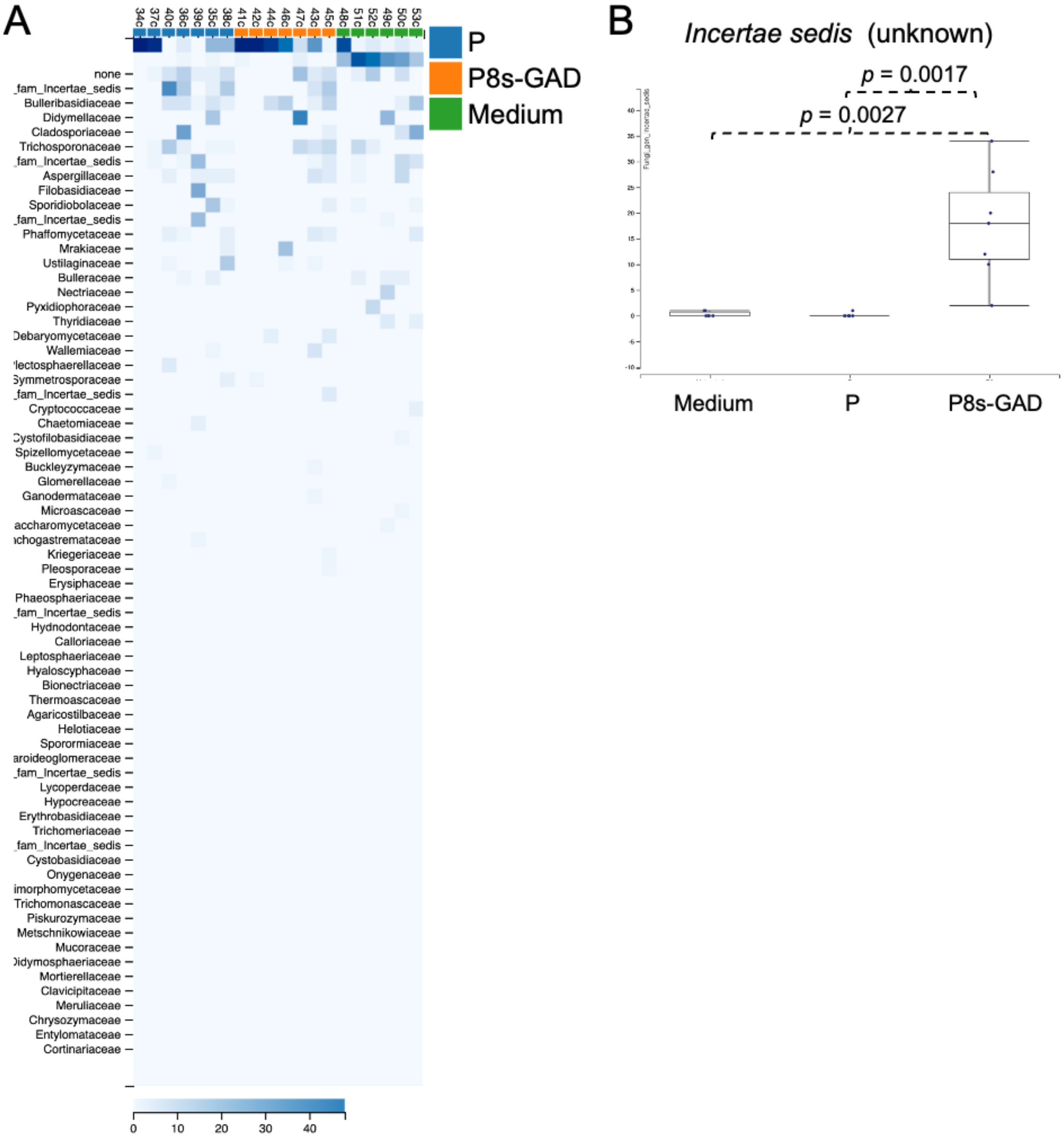

Supplemental Figure 16. A) Fungal family level in EAE mice treated with Medium, P-*L. lactis*, and P8s-GAD *L. lactis,* all supplemented with 200 mM GA, at D28. B) Abundance of unknown *Incertae sedis* at D28.

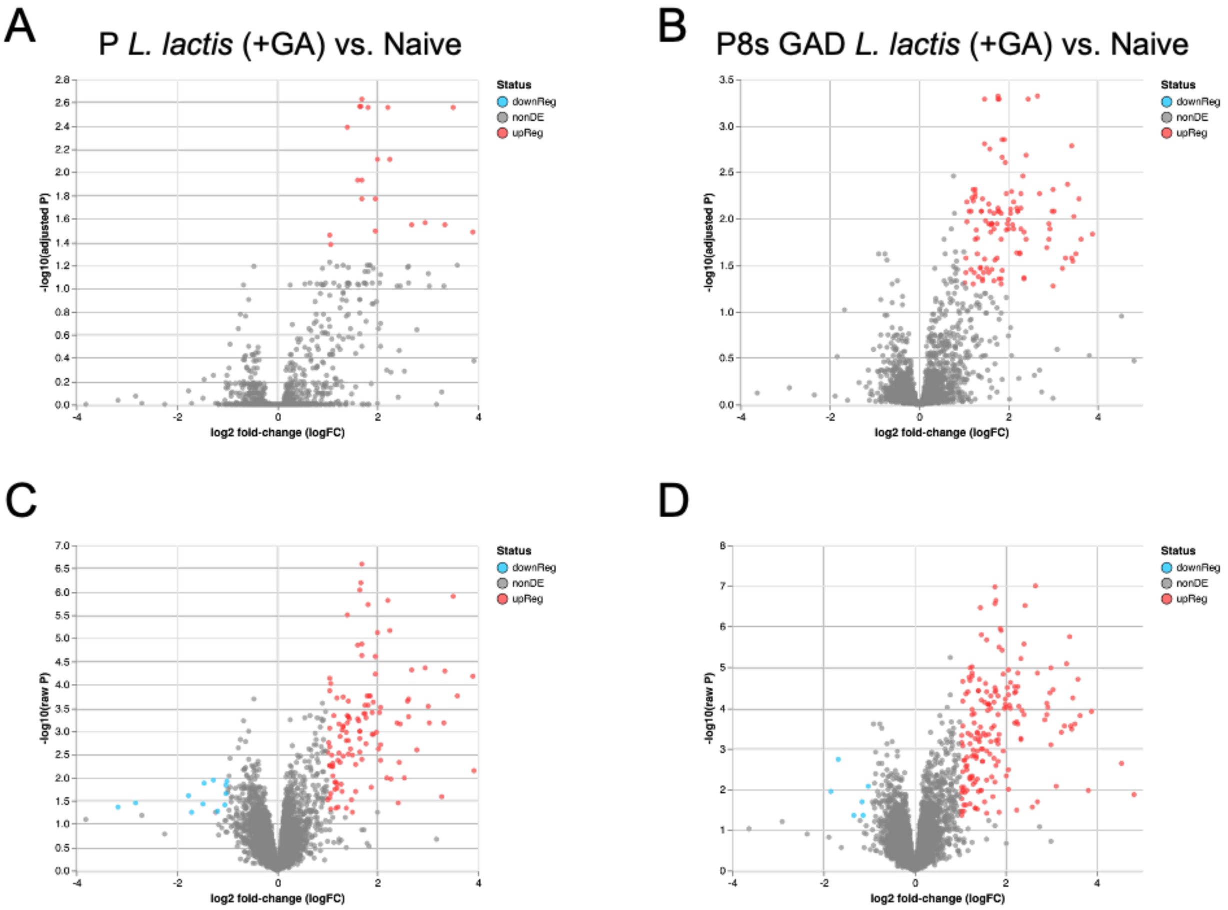

Supplemental Figure 17. Proteomics analysis of whole brains of EAE mice with P-*L. lactis* and P8s-GAD *L. lactis* supplemented with GA compared to naive. A-C) Volcano plots of the brain proteome of P-*L. lactis*-treated EAE mice compared to naïve mice, with proteins highlighted based on significant FDR value (A) and significant raw-p value (C); B-D) P8s-GAD-*L. lactis*-treated EAE mice compared to naïve mice with proteins highlighted based on significant FDR value (B) and significant raw-p value (D).
